# Disparities in cancer incidence, stage at diagnosis, treatment and mortality across socioeconomic groups in Finland: a register-based population study

**DOI:** 10.1136/bmjph-2025-002829

**Published:** 2025-10-22

**Authors:** Xianhua Zai, Peng Li, Luca Dei Bardi, Kaarina Korhonen, Margherita Moretti, Mikko Myrskylä, Pekka Martikainen

**Affiliations:** 1 Max-Planck-Institute for Demographic Research, Rostock, Germany; 2 Max Planck – University of Helsinki Center for Social Inequalities in Population Health, Rostock, Germany; 3 Helsinki Institute for Demography and Population Health, University of Helsinki, Helsinki, Finland

**Keywords:** Epidemiology, education, Incidence, Sociodemographic Factors

## Abstract

**Background:**

Socioeconomic inequalities in various cancer-related indicators exist globally, but the evidence is fragmented even within countries. We aim to assess disparities in cancer incidence, stage at diagnosis, treatment and mortality by socioeconomic status (SES, measured as education and income) in Finland across the entire cancer journey.

**Methods:**

Among the total Finnish population aged 30+ years, we identified those newly diagnosed with cancer between 2000–2019 based on Finnish Population Register and Cancer Register data. We calculated standardised rate ratios (SRRs) to compare incidence between SES groups. We used logistic regression models to estimate OR of being diagnosed with later stage cancer and of receiving specific treatments, and Cox models to estimate HR of mortality across SES groups.

**Findings:**

Among 4 536 724 Finnish residents, 240 746 men and 228 776 women were newly diagnosed with cancer between 2000 and 2019. Systematic disparities were observed for incidence, stage at diagnosis, treatment and mortality for all cancers and for most types of cancers. Among both men (SRR 1.17, 95% CI 1.16 to 1.19) and women (SRR 1.05, 95% CI 1.04 to 1.06), those with lower education had higher total cancer incidence rates than those with higher education. Patients with lower education had higher odds of being diagnosed with stage IV cancer than patients with higher education (men: OR 1.10, 95% CI 1.08 to 1.12; women: OR 1.15, 95% CI 1.13 to 1.17). Both male and female patients with lower education had a lower likelihood of being treated with surgery, chemotherapy and radiation than patients with higher education. Finally, patients with lower education had higher mortality rates than patients with higher education (men: HR 1.13, 95% CI 1.10 to 1.16; women: HR 1.14, 95% CI 1.11 to 1.17). Similar patterns were observed by income.

**Conclusions:**

Low SES individuals were disadvantaged in all aspects of their cancer journey for total cancer and most cancer types.

**WHAT IS ALREADY KNOWN ON THIS TOPIC**

- Socioeconomic inequalities in various cancer-related indicators exist, but the evidence is fragmented.
- A comprehensive understanding of disparities across socioeconomic groups throughout the whole cancer journey, including incidence, stage at diagnosis, treatment and mortality, is needed to guide cancer control practices.

**WHAT THIS STUDY ADDS**

- Finnish data covering the years 2000–2019 show that those with low socioeconomic status are disadvantaged in all aspects of their cancer journey, including later diagnosis, lower likelihood of treatment and higher mortality for most major cancer types.

**HOW THIS STUDY MIGHT AFFECT RESEARCH, PRACTICE OR POLICY**

- Our results underscore the crucial role of socioeconomic factors and call for policy interventions aimed at reducing inequalities across the whole cancer journey in Finland.

## Introduction

 Cancer is a complex disease characterised by disparities at the individual, societal and national levels.^1 2^ Social inequalities in cancer risks, diagnosis, treatment and survival have been studied and reported globally in recent decades,^3^,^16^ although the results have been fragmented and unsystematic. Disparities have been observed not only between countries, but also within high-income countries, despite their investments in social security and health insurance systems, and their widely accessible, high-quality healthcare.^17^,^21^ The European Union (EU) launched the ‘Europe’s Beating Cancer Plan’ in 2022, in which reducing cancer disparities across the EU is one of the key objectives.^22^ In addition to promoting healthy lifestyles, ensuring equal access to healthcare services and early cancer detection across socioeconomic status (SES) groups is essential to achieving this goal. Detailed knowledge of disparities in cancer stage at diagnosis, treatment received and survival is urgently needed.

Even in countries with universal healthcare systems, people with higher SES are more likely to receive cancer diagnoses at earlier stages, to undergo more comprehensive or radical cancer treatments, and to have better overall cancer survival rates.^923^,^25^ Studies from Nordic countries have found systematic socioeconomic inequalities in stage at diagnosis, treatment and survival for breast cancer, lung cancer, colorectal cancer and non-Hodgkin lymphoma.^26^ However, current knowledge about cancer-related disparities is fragmented, as prior studies mostly focused on isolated or incomplete aspects of cancer, including incidence, stage at diagnosis, treatment and/or survival. To guide cancer control practices, a more comprehensive understanding of inequalities throughout the whole cancer journey covering the entire population is needed.

In the context of Finland, previous studies have primarily focused on disparities in survival and cancer incidence across SES groups.^2127^,^35^ There is, however, no available evidence on national-level differences across SES groups in stage at diagnosis and treatment for all cancers or other important cancer types, such as lung cancer and melanoma. Thanks to the exhaustive Finnish register data, which cover the entire population over many decades, it is possible to track the complete cancer histories and survival outcomes of individuals, enabling research on SES disparities in the cancer journey without the limitations of self-report bias or loss to follow-up. This study aims to provide an integrated and detailed analysis of disparities across SES groups in various cancer-related aspects, thereby contributing to a more cohesive and comprehensive understanding of these inequalities and offering new insights for mitigating existing cancer inequalities in European countries.

## Materials and methods

### Study design

This study was based on the total population of Finland aged 30 years or older between 2000 and 2019, as shown in online supplemental figure 1. Information on gender, education level, income, marital status and origin background was extracted from the population register of Statistics Finland.

We identified the first primary cancer diagnoses between 2000 and 2019 using information on cancer site and morphology (according to the International Classification of Diseases for Oncology edition third, ICD-O-3) and cancer stage from the Finnish Cancer Registry (1953–2021). Non-melanoma skin cancer (C44) was excluded. We considered only the first cancer diagnosis for each individual from 1 January 2000 to the date of death, the date of emigration, or 31 December 2019, whichever came first. The survival status was followed until the date of death due to cancer, the date of emigration, 31 December 2021, or up to 10 years after the cancer diagnosis, whichever came first. Data on cancer-specific mortality were extracted from the Finnish Cancer Registry (1953–2021) and deaths due to other causes were censored. Data on anti-cancer treatments (surgery, radiotherapy, chemotherapy, including both intravenous and oral chemotherapies, and other therapies) were extracted from the Care Register of healthcare (1970–2021) maintained by the Finnish Institute for Health and Welfare and the Register of Prescription Medication Purchases (1995–2021) collected by the Social Insurance Institution of Finland. Individuals diagnosed with cancer before 1 January 2000 were excluded from the study. We analysed all cancers and the five most common site-specific cancers in men and women, respectively, including breast (ICD-O-3, C50), prostate (C61), colorectal (C18–C20), melanoma (C43), corpus uteri (C54) and lung cancers (C34), while grouping all other cancers into a residual category. All cancer diagnoses were included in the cancer incidence analyses, and cancer cases determined by death certificate or autopsy only (16 111 men, 11 089 women) were excluded from the stage, treatment and survival analyses (online supplemental figure 1). We focused on the population aged 30 years or older because cancer incidence is low among individuals under age 30, and the highest education level is generally stable after age 30.

### Definition of key covariates

Education level of patients with cancer was based on the highest attained qualification before the cancer diagnosis. We classified education level according to the International Standard Classification of Education (ISCED-2011) as low (ISCED 0–2: early childhood education, primary education and lower secondary education), medium (ISCED 3–4: upper secondary level and post-secondary non-tertiary education) or high (ISCED 5–8: short-cycle tertiary education, bachelor’s or equivalent level, master’s or equivalent level and doctoral or equivalent level). Information on disposable personal income was based on salary, self-employment and property income and current transfers received after taxes. We adjusted personal income for inflation and calculated the 5-year average before the year in which the individual was first diagnosed with cancer. In the analysis, income was classified as low, medium or high based on terciles within 5-year age groups. Other covariates included age at cancer diagnosis (linear, square and quadratic terms), year of diagnosis, year of birth, marital status (married, widowed, divorced and never married), origin (Finnish background: at least one parent born in Finland; foreign background: both parents born abroad), region of residence (a total of 19 regions) and urbanisation level of the municipality of residence (urban, semiurban and rural). For individuals diagnosed with cancer, all covariates were measured the year preceding the cancer diagnosis. For the incidence analysis, the exposure population was stratified by education/income level at each year and the total person-years were counted accordingly.

### Statistical methods

The characteristics of cancer cases were shown in the form of mean and SD or counts and percentages. For all cancers and the five most common cancer types in men and women, respectively, the age-standardised incidence rates (ASRs) were calculated for different SES groups using the European standard population (2013 edition).^36^ ASRs of low and medium SES groups were compared with ASRs of high SES groups using standardised rate ratios (SRRs) and the 95% CI.^37^ We used logistic regression models to estimate the OR of being diagnosed with stage IV compared with other cancer stages for low and medium SES groups compared with high SES groups. These models were adjusted for age at cancer diagnosis, year of diagnosis, year of birth, marital status, origin, region of residence and level of urbanisation of the municipality of residence, stratified by cancer type and gender. For the models including all cancers, cancer type was adjusted as a dummy factor. Individuals with missing information on the stage at diagnosis were excluded from the modelling (74 165 men, 66 671 women). Logistic regression models were used to estimate the OR of receiving a specific treatment (surgery, chemotherapy and radiation) compared with not receiving that treatment for high and medium SES groups compared with low SES groups, adjusted for covariates and stage at diagnosis (samples with missing information on the stage at diagnosis were included).

Cox proportional hazards regression was used to model HR for mortality across SES groups for all cancers and site-specific subtypes. Cox models were adjusted for age at diagnosis, year of diagnosis, stage at diagnosis, treatments, year of birth, marital status, origin, region of residence and level of urbanisation of the municipality of residence, stratified by cancer type and gender. For all cancer models, the cancer type was adjusted.

In order to distinguish the individual effects of education and income from each other, only education or income was included in the main model above (M1). In order to explore the confounding effects of education and income, we conducted sensitivity analyses by mutually adjusting education and income in the model (M2), and then further adding their interaction in the model (M3) to examine how the main effects changed from those in the main model (online supplemental figures 10–14). Because a large proportion (30%) of cancer diagnoses had missing information in the registry, we looked at the missing patterns by SES level in online supplemental table 5. All analyses were conducted using R (V.4.3.1).^38^

### Patient and public involvement

No patients were involved in defining the research question, or in the study design or implementation. However, attention to cancer disparities in the public media and discussions with patients with cancer helped to motivate the initiation of the study. Plain language messages about the results will be shared with the public.

## Results

### Sample characteristics

Online supplemental figure 1 describes the target population and number of subjects included in each analysis. Among 4 536 724 Finnish residents, a total of 469 522 population were newly diagnosed with cancer (240 746 men and 228 776 women) between 2000 and 2019. Table 1 describes the summary statistics for the subjects newly diagnosed with cancer, and for men and women separately. In terms of education levels, 50%, 27% and 23% of men were classified as having low, medium and high education, respectively, while the corresponding percentages for women were 46%, 29% and 25%. Based on income terciles by age group and calendar year, 23% of men and 35% of women were classified as having low income, while 44% of men and 28% of women were classified as having high income. The average age at diagnosis was 69 for men (SD=11) and 67 for women (SD=14). The most common cancer types were prostate (36%) and lung cancer (12%) for men and breast cancer (35%) and colorectal cancer (10%) for women. During the study period, 40% of men and 37% of women died of cancer.

**Table 1:** Characteristics of the study population: newly diagnosed cancer cases during 2000–2019

	Total	Men	Women
	N=469 522	N=240 746	N=228 776
Education (N, %)			
High	110 709 (23.6)	54 159 (22.5)	56 550 (24.7)
Medium	131 630 (28.0)	65 611 (27.3)	66 019 (28.9)
Low	227 183 (48.4)	120 976 (50.3)	106 207 (46.4)
Income (N, %)			
High	169 928 (36.2)	106 248 (44.1)	63 680 (27.8)
Medium	163 836 (34.9)	78 061 (32.4)	85 775 (37.5)
Low	135 758 (28.9)	56 437 (23.4)	79 321 (34.7)
Age at diagnosis (mean, SD)	68.17 (12.6)	68.88 (11.3)	67.42 (13.8)
Source of diagnoses (N, %)			
Clinical	442 322 (94.2)	224 635 (93.3)	217 687 (95.2)
Death certificate/autopsy only	27 200 (5.8)	16 111 (6.7)	11 089 (4.8)
Cancer types (N, %)*			
Bladder	9461 (2.0)	9461 (3.9)	NA
Breast	79 314 (16.9)	NA	79 314 (34.7)
Colorectum	47 069 (10.0)	24 191 (10.0)	22 878 (10.0)
Lung	41 884 (8.9)	28 367 (11.8)	13 517 (5.9)
Melanoma	19 676 (4.2)	10 211 (4.2)	9465 (4.1)
Prostate	85 727 (18.3)	85 727 (35.6)	NA
Corpus uteri	14 361 (3.1)	NA	14 361 (6.3)
Others	172 030 (36.6)	82 789 (34.4)	89 241 (39.0)
Cancer stage (N, %)			
Stage I-II	144 660 (30.8)	85 141 (35.4)	59 519 (26.0)
Stage III	133 234 (28.4)	64 325 (26.7)	68 909 (30.1)
Stage IV	50 792 (10.8)	17 115 (7.1)	33 677 (14.7)
Missing	140 836 (30.0)	74 165 (30.8)	66 671 (29.1)
Treatment (N, %)†			
Surgery	198 646 (42.3)	71 402 (29.7)	127 244 (55.6)
Radiation	87 481 (18.6)	37 877 (15.7)	49 604 (21.7)
Chemotherapy	90 528 (19.3)	36 596 (15.2)	53 932 (23.6)
Hormone therapy	96 240 (20.5)	42 327 (17.6)	53 913 (23.6)
Other therapies	21 083 (4.5)	11 240 (4.7)	9843 (4.3)
Outcome (N, %)‡			
Overall deaths	261 768 (55.8)	145 912 (60.6)	115 856 (50.6)
Cancer-specific deaths	179 785 (38.3)	95 850 (39.8)	83 935 (36.7)
Survival years (mean, SD)‡	5.05 (5.2)	4.69 (5.0)	5.43 (5.4)
Marital status (N, %)			
Married	258 410 (55.0)	153 965 (64.0)	104 445 (45.7)
Unmarried	62 015 (13.2)	31 703 (13.2)	30 312 (13.2)
Divorced	71 416 (15.2)	33 751 (14.0)	37 665 (16.5)
Widowed	77 681 (16.5)	21 327 (8.9)	56 354 (24.6)
Origin background (N, %)			
Finnish	460 401 (98.1)	236 522 (98.2)	223 879 (97.9)
Foreign	9121 (1.9)	4224 (1.8)	4897 (2.1)
Urbanisation			
Urban	295 269 (62.9)	146 016 (60.7)	149 253 (65.2)
Semiurban	80 200 (17.1)	42 621 (17.7)	37 579 (16.4)
Rural	94 053 (20.0)	52 109 (21.6)	41 944 (18.3)

* Non-melanoma skin cancer (C44) was excluded.

† Treatments are not mutually exclusive, so the percentages do not add up to 100%.

‡ Overall deaths and cancer-specific deaths were followed up until the end of 2020.

NA, not available.

The population was stratified by education/income levels, as shown in online supplemental tables 1 and 2. Excluding gender-specific cancer types, the most prevalent types were colorectal cancer (9%) and melanoma (6%) for the highly educated, and lung cancer (12%) and colorectal cancer (11%) for the low educated. Across all treatment types, the highly educated were always more likely to receive the treatment, regardless of whether it was surgery (high: 51%, low: 36%), radiation therapy (high: 25%, low: 14%), chemotherapy (high: 24%, low: 15%), hormone therapy (high: 24%, low: 18%) or other therapies (high: 5%, low: 4%). During the study period, 27% of the highly educated and 47% of the low educated died of cancer. Similar characteristics were observed across income levels.

### Incidence

**Figure 1:**
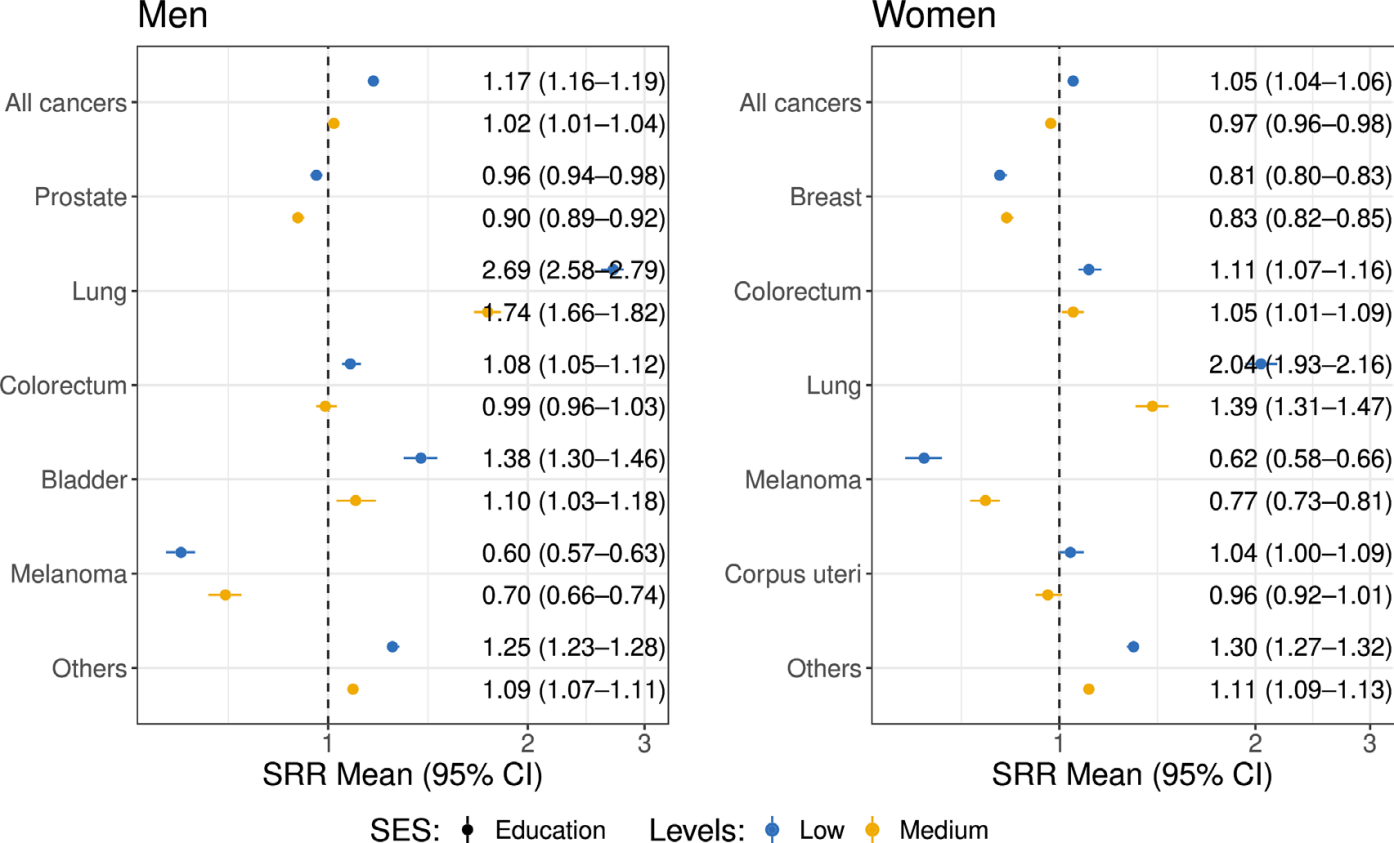
Standardised rate ratios (SRRs) of age standardised rates (ASRs) for different cancer types across education groups, stratified by sex. This figure shows the SRR between low (medium) education groups and high education groups. Dots with bars are the SRRs and the corresponding 95% CI of cancer incidences (ASRs) among men (women) with medium (yellow) and low (blue) education levels compared with those with high education levels. SES, socioeconomic status.

Among the total target population (2 221 291 men and 2 315 433 women), newly diagnosed cancer cases (240 746 men and 228 776 women; excluding non-melanoma skin cancer) were used to calculate cancer incidences across SES groups (online supplemental figure 1). Figure 1 illustrates the SRRs for cancer incidences (ASRs) for those with low (blue) and medium (yellow) education vs for those with high education by gender. Low education was generally associated with higher incidence of all cancer types in both men (SRR 1.17, 95% CI 1.16 to 1.19) and women (SRR 1.05, 95% CI 1.04 to 1.06). This pattern was consistent for lung, colorectal and ‘other’ cancers in both sexes. In contrast, the trend for melanoma (men SRR 0.60, 95% CI 0.57 to 0.63; women SRR 0.62, 95% CI 0.58 to 0.66), prostate cancer and breast cancer was reversed, with higher incidence being observed among those with higher education. Online supplemental figure 2 illustrates a similar trend for income. ASRs across SES groups and corresponding SRRs (low vs high, medium vs high), by sex, cancer type and cancer stage are shown in online supplemental tables 3and 4. Men and women with lower SES (educational attainment and income) generally had higher cancer incidence.

### Stage at diagnosis

**Figure 2:**
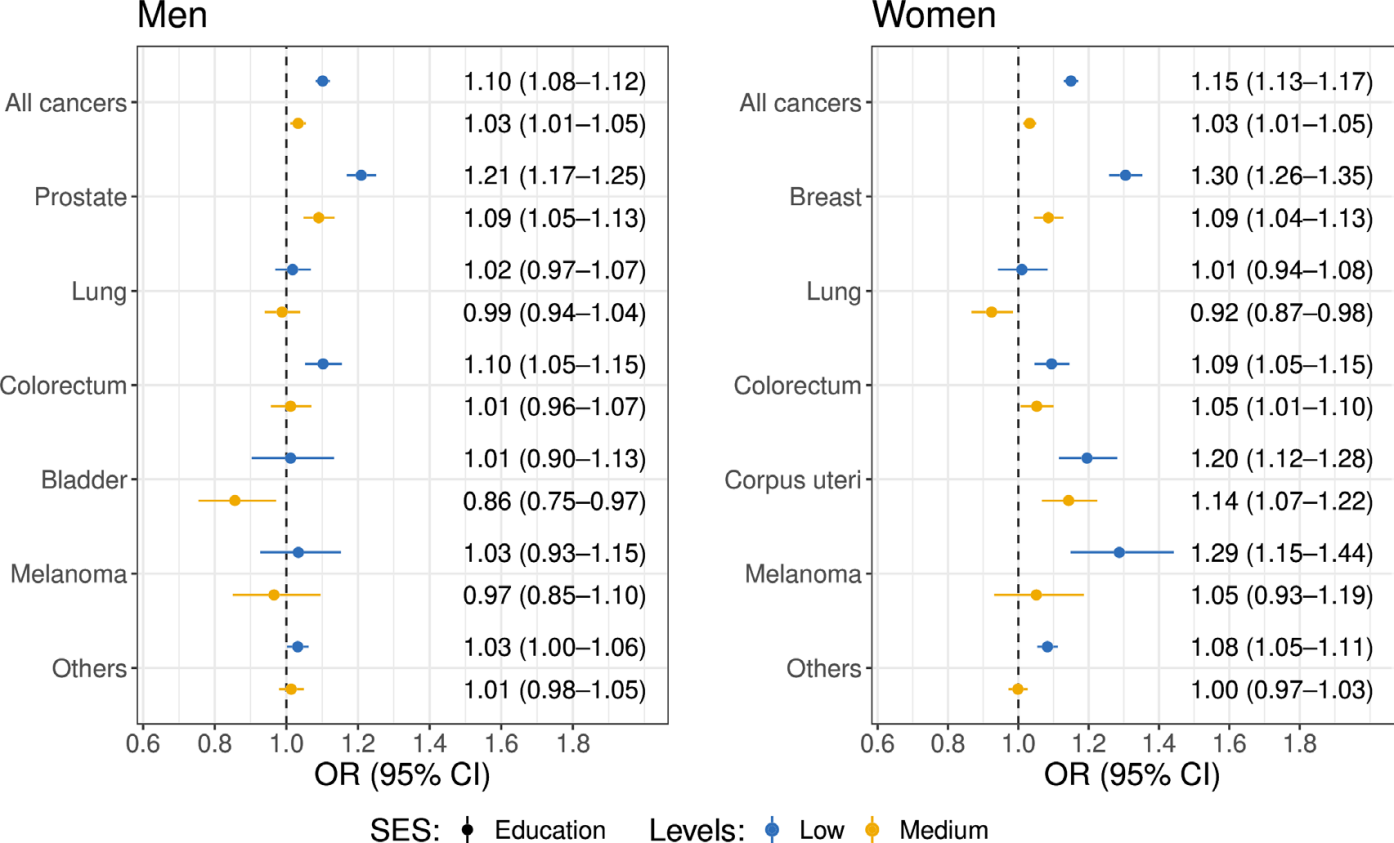
ORs of being diagnosed with stage IV cancer (ref. stage I–III cancer) across education groups by cancer type, stratified by sex. The models are adjusted for age at cancer diagnosis, year at diagnosis, year of birth, marital status, origin, region of residence, urbanisation of the region and education level stratified by cancer type. For all cancer models, cancer type is adjusted as a dummy factor. The dots with bars represent the ORs and corresponding 95% CIs for being diagnosed with stage IV cancer among individuals with low (blue) and medium (yellow) education levels compared with those with high education levels. SES, socioeconomic status.

The newly diagnosed cancer cases that were not identified from a death certificate or autopsy only and had no missing information on the stage (137 989 men and 155 409 women) were used to assess the associations between SES and cancer stage at diagnosis (online supplemental figure 1). Figure 2 shows that among both men and women, lower SES was generally associated with higher odds of being diagnosed with cancer at a later stage. Men with lower education had a higher likelihood of being diagnosed with stage IV of all cancers (OR 1.10, 95% CI 1.08 to 1.12), and specifically of prostate cancer (OR 1.21, 95% CI 1.17 to 1.25) and colorectal cancer (OR 1.10, 95% CI 1.05 to 1.15). Among women, lower SES was similarly linked to later diagnosis of all cancers (OR 1.15, 95% CI 1.13 to 1.17), and specifically of breast cancer (OR 1.30, 95% CI 1.26 to 1.35), colorectal cancer (OR 1.09, 95% CI 1.05 to 1.15), corpus uteri cancer (OR 1.20, 95% CI 1.12 to 1.28) and melanoma (OR 1.29, 95% CI 1.15 to 1.44). Similar patterns were observed across income levels (online supplemental figure 3).

### Treatment

For treatment with surgery, all solid cancer types were investigated, while lymphoma (C81–C86), multiple myeloma (C90) and leukaemia (C91–C95) were excluded, and a total of 122 252 men and 140 705 women were included in the models (online supplemental figure 1). For prostate cancer, surgery and radiation therapy were combined. For lung cancer, only non-small-cell lung cancer was included.

**Figure 3:**
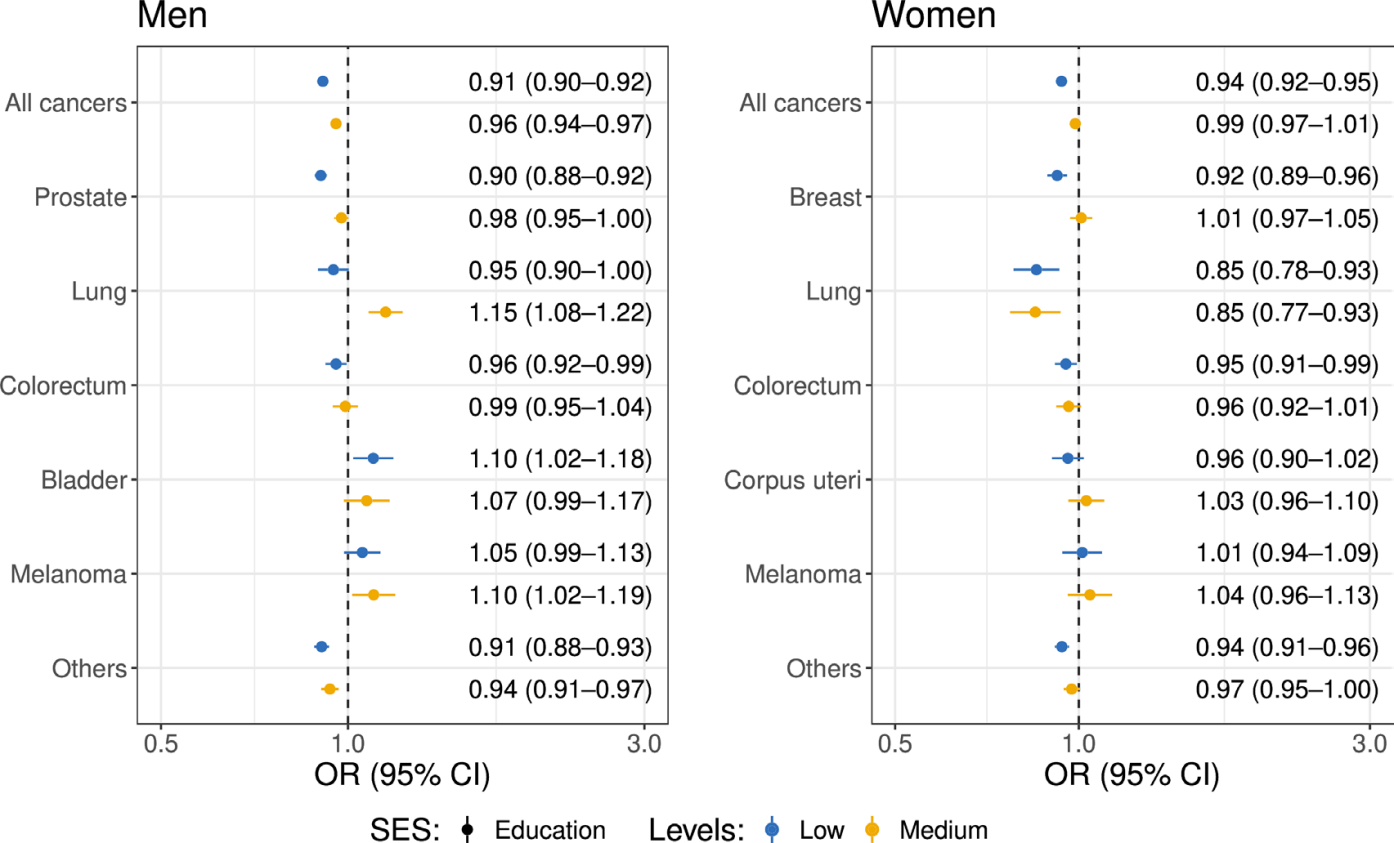
ORs of being treated with surgery (ref. no surgery) for solid cancers across education groups by cancer type, stratified by sex. All solid cancer types are included, while lymphoma (C81–C86), multiple myeloma (**C90**) and leukaemia (C91–C95) are excluded. The models are adjusted for stage at cancer diagnosis, age at diagnosis, year at diagnosis, year of birth, marital status, origin, region of residence, urbanisation of the region and education level stratified by cancer type. For prostate cancer, surgery and radiation therapy were combined. For lung cancer, only non-small-cell lung cancer is included. For all cancer models, cancer type is adjusted as a dummy factor. The dots with bars represent the OR and corresponding 95% CIs for being treated with surgery among individuals with medium (yellow) and low (blue) education levels compared with those with high education levels. For lung cancer specifically, only non-small-cell lung cancers were included. For prostate cancer, surgery and radiation were combined. SES, socioeconomic status.

Figure 3 shows that for both men and women, lower SES was generally associated with lower odds of being treated with surgery across different cancer stages. Among men, low education was consistently linked with a lower probability of undergoing surgery for all cancers (OR 0.91, 95% CI 0.90 to 0.92). Similar trends were observed across specific cancer types, but no significant differences were found for lung cancer and melanoma. Low education was associated with decreased odds of undergoing surgery for prostate cancer (surgery and radiation combined OR 0.90, 95% CI 0.88 to 0.92) and colorectal cancer (OR 0.96, 95% CI 0.92 to 0.99), but higher odds of undergoing surgery for bladder cancer (OR 1.10, 95% CI 1.02 to 1.18). Among women, low education was consistently linked with lower odds of undergoing surgery (figures3  4) for all cancers (OR 0.94, 95% CI 0.92 to 0.95), breast cancer (OR 0.92, 95% CI 0.89 to 0.96), lung cancer (OR 0.85, 95% CI 0.78 to 0.93) and colorectal cancer (OR 0.95, 95% CI 0.91 to 0.99), while no significant differences were found for corpus uteri cancer and melanoma. In the case of income levels among men (online supplemental figure 4), the SES differences were even larger than those for education, with men with low income having a lower likelihood of undergoing surgery than men with high income (OR 0.79, 95% CI 0.77 to 0.80). Similar patterns were observed across income levels among women, but for women, low income was also significantly associated with lower odds of undergoing surgery for corpus uteri cancer, whereas education was found to have a non-significant effect on this risk.

**Figure 4:**
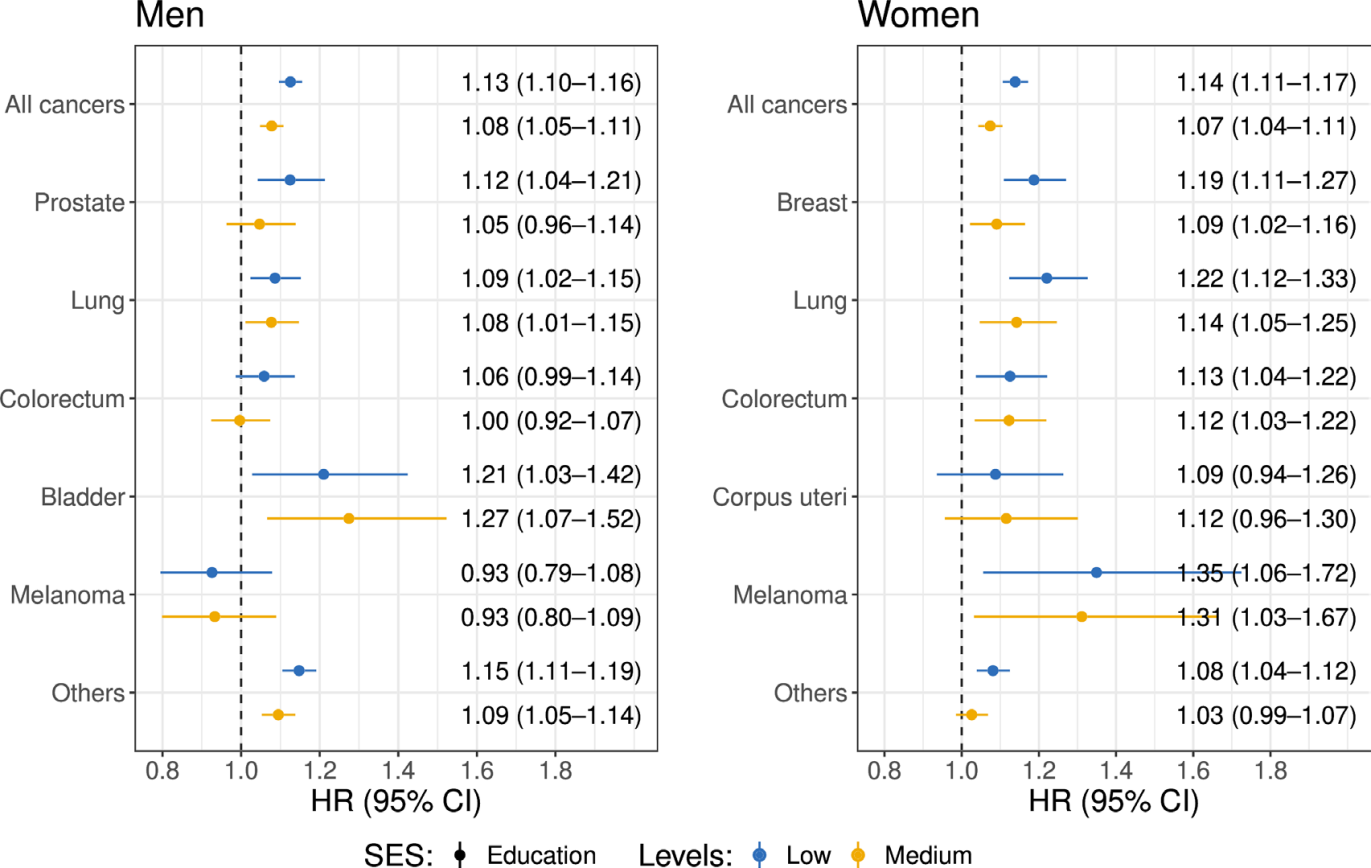
HRs of cancer-specific mortality across education groups by cancer type, stratified by sex. The models are adjusted for stage at cancer diagnosis, treatment, age at diagnosis, year at diagnosis, year of birth, marital status, origin, region of residence, urbanisation of the region and education level stratified by cancer type. For all cancer models, cancer type is adjusted as a dummy factor. The dots with bars represent the HR and corresponding 95% CIs for mortality among cancer patients with low (blue) and medium (yellow) education levels compared with those with high education levels. SES, socioeconomic status.

For other cancer therapies, a total of 137 989 men and 155 409 women were included in the models (online supplemental figure 1). Online supplemental figure 5 shows that lower education was linked to a lower likelihood of being treated with chemotherapy for men (OR 0.96, 95% CI 0.94 to 0.98) and women (OR 0.94, 95% CI 0.92 to 0.96). Stratified by cancer type, lower education among men was linked to a lower likelihood of undergoing chemotherapy for prostate cancer (OR 0.91, 95% CI 0.89 to 0.93), colorectal cancer (OR 0.90, 95% CI 0.87 to 0.95), and melanoma (OR 0.81, 95% CI 0.69 to 0.94), and to a lower likelihood of undergoing radiation therapy for prostate cancer (surgery and radiation combined OR 0.90, 95% CI 0.88 to 0.92), lung cancer (OR 0.94, 95% CI 0.90 to 0.99) and melanoma (OR 0.78, 95% CI 0.68 to 0.90). Among women, there were significant differences in the likelihood of undergoing chemotherapy treatment for specific cancer types (online supplemental figure 5) only for colorectal cancer (OR 0.87, 95% CI 0.83 to 0.92). Similarly, no significant associations were observed for the chances of undergoing radiation therapy (online supplemental figure 7), but the low educated had a lower likelihood of undergoing this treatment for breast cancer (OR 0.95, 95% CI 0.92 to 0.98) and melanoma (OR 1.36, 95% CI 1.13 to 1.65). Online supplemental figure 6 shows that individuals with low income had a significantly lower likelihood of undergoing chemotherapy for all specific cancer types identified, but especially for bladder cancer and melanoma in men and corpus uteri and melanoma in women. Low income was not associated with lower rates of radiation therapy in men (online supplemental figure 8), but results for specific cancer types showed that men with low income had a lower likelihood of undergoing radiation therapy, with the exception that they were more likely to be treated with radiation for melanoma and other cancers. Among women, low income was associated with a lower likelihood of undergoing chemotherapy for all cancers and for breast, lung and colorectal cancer.

### Survival

A total of 137 989 men and 155 409 women were included in the Cox models (online supplemental figure 1). Figure 4 indicates that lower SES was generally associated with higher mortality for all cancer types. Among men, lower education was consistently associated with higher mortality risk for all cancers (HR 1.13, 95% CI 1.10 to 1.16) and most site-specific cancer types, including prostate cancer (HR 1.12, 95% CI 1.04 to 1.21), bladder cancer (HR 1.21, 95% CI 1.03 to 1.42) and other cancers (HR 1.15, 95% CI 1.11 to 1.19). Lower-educated women also had increased mortality risk for all cancers (HR 1.14, 95% CI 1.11 to 1.17) and for most site-specific cancer types. Lower education was associated with higher mortality risk for breast cancer (HR 1.19, 95% CI 1.11 to 1.27), lung cancer (HR 1.22, 95% CI 1.12 to 1.33), colorectal cancer (HR 1.13, 95% CI 1.04 to 1.22) and melanoma (HR 1.35, 95% CI 1.06 to 1.72). Similar patterns were observed across income levels among men and women (online supplemental figure 9).

### Sensitivity analysis

While education and income were adjusted in the model, as shown in M2 in online supplemental figure 10–14, the main effects of education and income were generally similar to and in the same direction as those when only one SES measure was included in the model (M1). Even when the interaction term for education and income was included in the model (M3), the main effects remained comparable to those from M1.

The results of the sensitivity analysis of missing information on stage at diagnosis by cancer type are shown in online supplemental table 5. For all cancers, no significant difference in missing rate on cancer stage across education levels was observed for both men (education OR 1.01, 95% CI 1.00 to 1.03) and women (education OR 1.01, 95% CI 0.99 to 1.02). Patterns similar to those for all cancers were observed for most site-specific cancers except melanoma in men (education OR 0.90, 95% CI 0.84 to 0.97) and corpus uteri in women (education OR 0.93, 95% CI 0.88 to 0.98). For all cancers, low income was associated with higher chances of having missing information on stage at diagnosis among both men (income OR 1.08, 95% CI 1.06 to 1.10) and women (income OR 1.05, 95% CI 1.03 to 1.07). Stronger patterns were observed for prostate, lung and colorectal cancer in men and breast and lung cancer in women, while opposite patterns were observed for melanoma in both men and women.

## Discussion

The findings indicate that men and women with lower SES (educational attainment and income) typically had higher cancer incidence, were more likely to be diagnosed at a later stage, were less likely to get treated and had higher mortality. Nevertheless, variations by cancer type and treatment type emerged.

Men and women with lower SES had much higher incidences of lung cancer and colorectal cancer than people with higher SES. Disparities in incidence were more inconsistent or reversed for melanoma, breast and prostate cancer. Men with lower SES had a higher probability of being diagnosed at a later stage of prostate cancer, colorectal cancer or other cancers, while women with lower SES were more likely to be diagnosed at a later stage of breast cancer, melanoma or corpus uteri cancer.

These findings align with those from a prior study that analysed population-based cancer registry data from the USA (1973–2001), which showed that lower education and income levels were associated with late stage diagnoses, particularly for breast and prostate cancers.^7^ Smaller clinical case studies have observed similar patterns.^5 6 23^ The differentials in the stage at which cancer is diagnosed might be related to the levels of accessibility, awareness or participation in cancer screening programmes among different SES groups, particularly those for breast, colorectal and prostate cancers. A study of breast cancer screening in the Netherlands found that women with medium and high levels of education had, respectively, 50% and 80% higher chances of receiving mammography screening than lower-educated women.^39^ Similar breast cancer screening patterns have been observed in the USA.^40^ The case of prostate cancer is more complex. As there are debates about the benefits and costs of screening in the general population, screening recommendations vary considerably by country, and evidence of overdiagnosis and overtreatment has been reported in Europe. Nonetheless, screening uptake has been shown to be lower among patients with low SES in different healthcare settings.^41 42^ Although participation rates in colorectal cancer screening programmes vary worldwide, population groups with low SES consistently have lower adherence within each country.^43 44^ In Finland, national screening programmes during the study period covered only breast and cervical cancers, so SES differences in screening participation cannot explain inequalities in other cancer types. However, broader socioeconomic disparities in healthcare access may contribute. Higher educated and higher income individuals are more likely to use occupational or private healthcare enabling faster access and broader choice of providers, while those with lower SES are more likely to forgo care or rely solely on public services facing longer waiting times and fewer options.^45 46^

In our study, we found that men with lower SES were less likely to receive specific oncological treatment, particularly surgery and radiation therapy if they had prostate, lung or bladder cancer. Among women, lower SES was similarly shown to be associated with a lower likelihood of receiving specific treatment across all cancer stages, especially for corpus uteri, breast or colorectal cancer. These findings are consistent with previous research on prostate,^8^ breast^23 47 48^ and colorectal cancers.^49^

In terms of survival, lower SES was found to be most often associated with higher mortality across all cancer types for both men and women, and particularly for prostate, breast and colorectal cancers. In addition, although populations with low SES had lower incidence rates of prostate cancer, breast cancer and melanoma, their mortality risk was much higher than that of the high SES group, possibly due to late diagnosis, poorer treatment and/or treatment adherence. These survival patterns are consistent with the findings from studies conducted in Southeast England for all cancer types,^13^ in Canada for all cancers,^9^ and for specific cancers like prostate,^13^ breast^23^ and lung cancers.^21^

Research that systematically assesses the SES disparities in cancer incidence, stage at diagnosis, treatment and survival in the total general population is essential for public health practices, but the evidence from previous studies has been insufficient. Some studies focused on individual aspects of cancer care, including incidence, stage at diagnosis, treatment or survival in the total general population.^50^,^52^ Other studies investigated two aspects of cancer care, such as incidence/risk and mortality or cancer treatment and survival.^35 53^ Only one study investigated cancer stage, treatment and survival simultaneously in the Osaka region of Japan and observed that the population living in high-deprivation areas had lower chances of early-stage detection, receipt of surgery and survival.^54^ Our research filled this gap by examining patterns across the entire cancer journey, from incidence and diagnosis to treatment and survival outcomes, providing evidence that can be used to develop cancer control strategies and mitigate health inequalities. Moreover, our study benefited from the use of data from the Finnish Cancer Registry, which stands out for its comprehensive coverage and detailed information from 1952 onward. Such longitudinal data linked to high-quality patient-level education and income data from the Population Registry are not available in many other contexts. By contrast, the measurements of SES factors used in previous research were diverse and were sometimes based on regional aggregated data only. For example, previous studies used indirect SES measures such as deprivation indexes,^8 54^ Medicaid enrolment and census tract poverty levels,^48^ postal code average income^14 23^ and average household income at the neighbourhood level.^49^

Nevertheless, this research had several limitations that should be considered when interpreting the findings. One key limitation was the lack of data on the mechanisms associated with cancer risk, such as lifestyle factors, environmental exposures and biological susceptibilities. Additionally, the study did not account for the potential impact of having other chronic diseases on cancer risks and treatments. For example, patients with low SES might have had more health conditions than the high SES groups, which could have affected their treatment decisions and efficacy, and thus also their survival chances. Another limitation, although common in many studies, was related to the measurement of SES. While personal income was used in this study, household income might be a better indicator of available material resources. However, this indicator is often unavailable for institutionalised individuals, who are typically older and at higher risk of cancer. Moreover, there was a high proportion of missing information in the Finnish Cancer Registry for the stage at diagnosis (30%), which is common in cancer registries. We analysed patterns of missing information on cancer stage across SES groups for overall cancers and specific cancer types. No significant difference in the missing rate was observed across education groups for overall cancers and most specific cancers, such as prostate, breast and lung cancers. However, disparities in missing stage observed across income groups for overall and specific cancers need further investigations. Moreover, the relationship between education and income was complex and not easy to disentangle. We performed sensitivity analyses to test whether educational attainment and income captured the same SES dynamics, first by including both SES factors, and then by including the interaction terms in the models. The main effects of education and income were relatively stable while they were mutually adjusted with interactions, which indicated that education and income alone could not fully capture the information of SES and further investigation is needed. Finally, we did not have detailed information on the type of treatment, such as whether it was a radical or an adjuvant treatment.

The finding that lower SES is often correlated with later diagnosis, a lower likelihood of treatment and higher mortality underscores the crucial role of SES when cancer is diagnosed, how it is treated and, ultimately, what the chances of survival are. This evidence calls for policy interventions aimed at reducing SES-related disparities across the whole cancer journey. Two of the most likely potential mechanisms could relate to differences in the levels of participation in screening programmes or access to healthcare more generally, and differences in behaviours across SES groups. Lifestyle factors such as diet, exercise, smoking and alcohol consumption are often correlated with SES and may significantly mediate the relationship between SES and cancer outcomes. As these are avoidable behavioural pathways, policy interventions could include providing enhanced and targeted screening and early detection programmes for lower SES subgroups, improving access to quality treatment regardless of income or education level, promoting healthier lifestyles through education and community programmes, and increasing cancer literacy through education campaigns. Addressing these disparities has the potential not only to improve health outcomes for marginalised groups, but also to contribute to the broader goals of achieving equity in healthcare and overall improvements in public health, which would benefit society as a whole.

## Supplementary material

10.1136/bmjph-2025-002829 online supplemental file 1

## References

[1] International Agency for Research on Cancer (2019). Reducing social inequalities in cancer: evidence and priorities for research.

[2] McCormack V, Newton R, International Agency for Research on Cancer (2019). Research priorities for social inequalities in cancer in sub-saharan africa.

[3] Cella DF, Orav EJ, Kornblith AB (1991). Socioeconomic status and cancer survival. J Clin Oncol.

[4] Schrijvers CT, Mackenbach JP, Lutz JM (1995). Deprivation, stage at diagnosis and cancer survival. Int J Cancer.

[5] Macleod U, Ross S, Gillis C (2000). Socio-economic deprivation and stage of disease at presentation in women with breast cancer. Ann Oncol.

[6] Dalton SO, Düring M, Ross L (2006). The relation between socioeconomic and demographic factors and tumour stage in women diagnosed with breast cancer in Denmark, 1983-1999. Br J Cancer.

[7] Clegg LX, Reichman ME, Miller BA (2009). Impact of socioeconomic status on cancer incidence and stage at diagnosis: selected findings from the surveillance, epidemiology, and end results: National Longitudinal Mortality Study. Cancer Causes Control.

[8] Lyratzopoulos G, Barbiere JM, Greenberg DC (2010). Population based time trends and socioeconomic variation in use of radiotherapy and radical surgery for prostate cancer in a UK region: continuous survey. BMJ.

[9] Booth CM, Li G, Zhang-Salomons J (2010). The impact of socioeconomic status on stage of cancer at diagnosis and survival: a population-based study in Ontario, Canada. Cancer.

[10] Chornokur G, Dalton K, Borysova ME (2011). Disparities at presentation, diagnosis, treatment, and survival in African American men, affected by prostate cancer. Prostate.

[11] Danzig MR, Weinberg AC, Ghandour RA (2014). The association between socioeconomic status, renal cancer presentation, and survival in the United States: a survival, epidemiology, and end results analysis. Urology.

[12] Long B, Chang J, Ziogas A (2015). Impact of race, socioeconomic status, and the health care system on the treatment of advanced-stage ovarian cancer in California. Am J Obstet Gynecol.

[13] Klein J, von dem Knesebeck O (2015). Socioeconomic inequalities in prostate cancer survival: A review of the evidence and explanatory factors. Soc Sci Med.

[14] Filipe MD, Siesling S, Vriens MR (2021). The association of socioeconomic status on treatment strategy in patients with stage I and II breast cancer in the Netherlands. Breast Cancer Res Treat.

[15] Bryere J, Tron L, Menvielle G (2019). The respective parts of incidence and lethality in socioeconomic differences in cancer mortality. An analysis of the French network Cancer registries (FRANCIM) data. Int J Equity Health.

[16] Derette K, Rollet Q, Launay L (2022). Evolution of socioeconomic inequalities in cancer incidence between 2006 and 2016 in France: a population-based study. Eur J Cancer Prev.

[17] Singh G, Miller BA, Hankey BF (1975). Area Socioeconomic Variations in U.S. Cancer Incidence, Mortality, Stage, Treatment, and Survival, 1975–1999. Vol. NIH Publication No. 03-0000.

[18] Gupta S, Wilejto M, Pole JD (2014). Low Socioeconomic Status Is Associated with Worse Survival in Children with Cancer: A Systematic Review. PLoS ONE.

[19] Boscoe FP, Henry KA, Sherman RL (2016). The relationship between cancer incidence, stage and poverty in the United States. Int J Cancer.

[20] Zavala VA, Bracci PM, Carethers JM (2021). Cancer health disparities in racial/ethnic minorities in the United States. Br J Cancer.

[21] Vaccarella S, Georges D, Bray F (2023). Socioeconomic inequalities in cancer mortality between and within countries in Europe: a population-based study. Lancet Reg Health Eur.

[22] European Commission (2021). Europe’s beating cancer plan. https://commission.europa.eu/strategy-and-policy/priorities-2019-2024/promoting-our-european-way-life/european-health-union/cancer-plan-europe_en.

[23] Kumachev A, Trudeau ME, Chan KKW (2016). Associations among socioeconomic status, patterns of care and outcomes in breast cancer patients in a universal health care system: Ontario’s experience. Cancer.

[24] Mackillop WJ, Zhang-Salomons J, Groome PA (1997). Socioeconomic status and cancer survival in Ontario. J Clin Oncol.

[25] Adam M, Rueegg CS, Schmidlin K (2016). Socioeconomic disparities in childhood cancer survival in Switzerland. Int J Cancer.

[26] Ammitzbøll G, Levinsen AKG, Kjær TK (2022). Socioeconomic inequality in cancer in the Nordic countries. A systematic review. Acta Oncol.

[27] Lehto U-S, Ojanen M, Dyba T (2006). Baseline psychosocial predictors of survival in localised breast cancer. Br J Cancer.

[28] Kilpeläinen TP, Talala K, Raitanen J (2016). Prostate Cancer and Socioeconomic Status in the Finnish Randomized Study of Screening for Prostate Cancer. Am J Epidemiol.

[29] Gadeyne S, Menvielle G, Kulhanova I (2017). The turn of the gradient? Educational differences in breast cancer mortality in 18 European populations during the 2000s. Int J Cancer.

[30] Seikkula HA, Kaipia AJ, Ryynänen H (2018). The impact of socioeconomic status on stage specific prostate cancer survival and mortality before and after introduction of PSA test in Finland. Int J Cancer.

[31] Lehto U-S, Ojanen M, Väkevä A (2019). Early quality-of-life and psychological predictors of disease-free time and survival in localized prostate cancer. Qual Life Res.

[32] Savijärvi S, Seppä K, Malila N (2019). Trends of colorectal cancer incidence by education and socioeconomic status in Finland. Acta Oncol.

[33] Kilpeläinen TP, Talala K, Taari K (2020). Patients’ education level and treatment modality for prostate cancer in the Finnish Randomized Study of Screening for Prostate Cancer. Eur J Cancer.

[34] Finke I, Seppä K, Malila N (2021). Educational inequalities and regional variation in colorectal cancer survival in Finland. Cancer Epidemiol.

[35] (2021). Cancer in Finland 2019.

[36] European Union (2013). Revision of the european standard population.

[37] Silva S (1999). Cancer Epidemiology: Principles and Methods.

[38] R Core Development Team (2023). R: A Language and Environment for Statistical Computing.

[39] Aarts MJ, Voogd AC, Duijm LEM (2011). Socioeconomic inequalities in attending the mass screening for breast cancer in the south of the Netherlands—associations with stage at diagnosis and survival. Breast Cancer Res Treat.

[40] Elewonibi BR, Thierry AD, Miranda PY (2018). Examining Mammography Use by Breast Cancer Risk, Race, Nativity, and Socioeconomic Status. J Immigrant Minority Health.

[41] Ross LE, Taylor YJ, Howard DL (2011). Trends in prostate-specific antigen test use, 2000-2005. Public Health Rep.

[42] Williams N, Hughes LJ, Turner EL (2011). Prostate-specific antigen testing rates remain low in UK general practice: a cross-sectional study in six English cities. BJU Int.

[43] Klerk CM, Gupta S, Dekker E (2018). Expert Working Group ‘Coalition to reduce inequities in colorectal cancer screening’ of the World Endoscopy Organization. Socioeconomic and ethnic inequities within organised colorectal cancer screening programmes worldwide. Gut.

[44] Gini A, Jansen EEL, Zielonke N (2020). Impact of colorectal cancer screening on cancer-specific mortality in Europe: A systematic review. Eur J Cancer.

[45] OECD (2017). Finland: country health profile 2017. https://www.oecd.org/en/publications/finland-country-health-profile-2017_9789264283367-en.html.

[46] Blomgren J, Virta LJ (2020). Socioeconomic differences in use of public, occupational and private health care: A register-linkage study of a working-age population in Finland. PLoS ONE.

[47] Sail K, Franzini L, Lairson D (2012). Differences in treatment and survival among African-American and Caucasian women with early stage operable breast cancer. Ethn Health.

[48] Dreyer MS, Nattinger AB, McGinley EL (2018). Socioeconomic status and breast cancer treatment. Breast Cancer Res Treat.

[49] Dik VK, Aarts MJ, Van Grevenstein WMU (2014). Association between socioeconomic status, surgical treatment and mortality in patients with colorectal cancer. Br J Surg.

[50] Hussain SK, Altieri A, Sundquist J (2008). Influence of education level on breast cancer risk and survival in Sweden between 1990 and 2004. Int J Cancer.

[51] Abdoli G, Bottai M, Moradi T (2014). Cancer mortality by country of birth, sex, and socioeconomic position in Sweden, 1961-2009. PLoS ONE.

[52] Hemminki K, Li X (2003). Level of education and the risk of cancer in Sweden. Cancer Epidemiol Biomarkers Prev.

[53] Hastert TA, Beresford SAA, Sheppard L (2015). Disparities in cancer incidence and mortality by area-level socioeconomic status: a multilevel analysis. J Epidemiol Community Health.

[54] Odani S, Tabuchi T, Nakaya T (2023). Socioeconomic disparities in cancer survival: Relation to stage at diagnosis, treatment, and centralization of patients to accredited hospitals, 2005-2014, Japan. Cancer Med.

