# Supplementary material for: Disparities in cancer incidence, stage at diagnosis, treatment and mortality across socioeconomic groups in Finland: a register-based population study
Source: BMJ Public Health. 2025 Oct 22;3(2):e002829. doi: 10.1136/bmjph-2025-002829 (PMC12551501; doi:10.1136/bmjph-2025-002829)
Supplement: online supplemental file 1 [file bmjph-3-2-s001.docx]

Contents

[Supplementary Table 1: Characteristics of the study population: newly diagnosed cancer cases during 2000-2019 by education 3](#_Toc207126408)

[Supplementary Table 2: Characteristics of the study population: newly diagnosed cancer cases during 2000-2019 by income 5](#_Toc207126409)

[Supplementary Table 3: Age standardized cancer incidence rates (ASRs) and standardized rate ratios (SRRs) by cancer type and by stage across education groups, stratified by sex 7](#_Toc207126410)

[Supplementary Table 4: Age standardized cancer incidence rates (ASRs) and standardized rate ratios (SRRs) by cancer type and by stage across income groups, stratified by sex 9](#_Toc207126411)

[Supplementary Table 5: Patterns of missing information on stage at diagnosis by education and income 11](#_Toc207126412)

[Supplementary Figure 1. Study design and flow diagram 13](#_Toc207126413)

[Supplementary Figure 2. Standardized rate ratios (SRRs) of age standardized rates (ASRs) for different cancer types across income groups, stratified by sex 14](#_Toc207126414)

[Supplementary Figure 3. Odds ratios (OR) of being diagnosed with stage IV cancer (ref. stage I-II-III cancer) across income groups by cancer type, stratified by sex 15](#_Toc207126415)

[Supplementary Figure 4. Odds ratios (OR) of being treated with surgery (ref. no surgery) for solid cancers across income groups by cancer type, stratified by sex 16](#_Toc207126416)

[Supplementary Figure 5. Odds ratios (OR) of being treated with chemotherapy (ref. no chemotherapy) across education groups by cancer type, stratified by sex 17](#_Toc207126417)

[Supplementary Figure 6. Odds ratios (OR) of being treated with chemotherapy (ref. no chemotherapy) across income groups by cancer type, stratified by sex 18](#_Toc207126418)

[Supplementary Figure 7. Odds ratios (OR) of being treated with radiation therapy (ref. no radiation therapy) across education groups by cancer type, stratified by sex 19](#_Toc207126419)

[Supplementary Figure 8. Odds ratios (OR) of being treated with radiation therapy (ref. no radiation therapy) across income groups by cancer type, stratified by sex 20](#_Toc207126420)

[Supplementary Figure 9. Hazard Ratio (HR) of cancer-specific mortality across income groups by cancer type, stratified by sex 21](#_Toc207126421)

[Supplementary Figure 10: Sensitivity analyses of SES factors on being diagnosed with stage IV cancer for all cancers, stratified by sex 22](#_Toc207126422)

[Supplementary Figure 11: Sensitivity analyses of SES factors on treatment (surgery) for all cancers, stratified by sex 23](#_Toc207126423)

[Supplementary Figure 12: Sensitivity analyses of SES factors on treatment (chemotherapy) for all cancers, stratified by sex 24](#_Toc207126424)

[Supplementary Figure 13: Sensitivity analyses of SES factors on treatment (radiation therapy) for all cancers, stratified by sex 25](#_Toc207126425)

[Supplementary Figure 14: Sensitivity analyses of SES factors on cancer-specific mortality for all cancers, stratified by sex 26](#_Toc207126426)

### Supplementary Table 1: Characteristics of the study population: newly diagnosed cancer cases during 2000-2019 by education

|  | **High education** | **Medium education** | **Low education** |
| --- | --- | --- | --- |
|  | **N = 110,709** | **N = 131,630** | **N = 227,183** |
| **Income (N, %)** |  |  |  |
| High | 76,751 (69.3%) | 41,672 (31.7%) | 51,505 (22.7%) |
| Medium | 23,659 (21.4%) | 51,944 (39.5%) | 88,233 (38.8%) |
| Low | 10,299 (9.3%) | 38,014 (28.9%) | 87,445 (38.5%) |
| **Gender (N, %)** |  |  |  |
| Men | 54,159 (48.9%) | 65,611 (49.8%) | 120,976 (53.3%) |
| Women | 56,550 (51.1%) | 66,019 (50.2%) | 106,207 (46.7%) |
| **Age at diagnosis (mean, SD)** | 63.48 (12.8) | 63.84 (12.2) | 72.97 (10.9) |
| **Source of diagnoses (N, %)** |  |  |  |
| Clinical | 107,164 (96.8%) | 125,804 (95.6%) | 20,9354 (92.2%) |
| Death certificate only/Autopsy | 3,545 (3.2%) | 5,826 (4.4%) | 17,829 (7.8%) |
| **Cancer types (N, %)** ^a^ |  |  |  |
| Bladder | 1,806 (1.6%) | 2,299 (1.7%) | 5,356 (2.4%) |
| Breast | 25,908 (23.4%) | 25,321 (19.2%) | 28,085 (12.4%) |
| Colorectum | 10,196 (9.2%) | 12,635 (9.6%) | 24,238 (10.7%) |
| Lung | 5,095 (4.6%) | 10,543 (8.0%) | 26,246 (11.6%) |
| Melanoma | 6,955 (6.3%) | 5,974 (4.5%) | 6,747 (3.0%) |
| Prostate | 21,640 (19.5%) | 22,134 (16.8%) | 41,953 (18.5%) |
| Uteri | 3,371 (3.0%) | 4,240 (3.2%) | 6,750 (3.0%) |
| Others | 35,738 (32.3%) | 48,484 (36.8%) | 87,808 (38.7%) |
| **Cancer stage (N, %)** |  |  |  |
| Missing | 34,002 (30.7%) | 40,405 (30.7%) | 70,253 (30.9%) |
| Stage I-II | 35,647 (32.2%) | 38,145 (29.0%) | 59,442 (26.2%) |
| Stage III | 13,893 (12.5%) | 15,648 (11.9%) | 21,251 (9.4%) |
| Stage IV | 27,167 (24.5%) | 37,432 (28.4%) | 76,237 (33.6%) |
| **Treatment (N, %)** ^b^ |  |  |  |
| Surgery | 56,606 (51.1%) | 61,259 (46.5%) | 80,781 (35.6%) |
| Radiation | 27,073 (24.5%) | 28,992 (22.0%) | 31,416 (13.8%) |
| Chemotherapy | 26,009 (23.5%) | 30,877 (23.5%) | 33,642 (14.8%) |
| Hormone therapy | 26,960 (24.4%) | 27,369 (20.8%) | 41,911 (18.4%) |
| Other therapies | 5,299 (4.8%) | 6,726 (5.1%) | 9,058 (4.0%) |
| **Outcome (N, %)** ^c^ |  |  |  |
| Over-all deaths | 42,086 (38.0%) | 61,426 (46.7%) | 158,256 (69.7%) |
| Cancer specific deaths | 29,540 (26.7%) | 44,709 (34.0%) | 105,536 (46.5%) |
| **Survival years (mean, SD)** ^c^ | 5.94 (5.3) | 5.34 (5.2) | 4.45 (5.1) |
| **Marital status (N, %)** |  |  |  |
| Married | 72,738 (65.7%) | 73,217 (55.6%) | 112,455 (49.5%) |
| Unmarried | 13,701 (12.4%) | 20,702 (15.7%) | 27,612 (12.2%) |
| Divorced | 15,301 (13.8%) | 23,548 (17.9%) | 32,567 (14.3%) |
| Widowed | 8,969 (8.1%) | 14,163 (10.8%) | 54,549 (24.0%) |
| **Origin (N, %)** |  |  |  |
| Natives | 108,450 (98.0%) | 129,474 (98.4%) | 222,477 (97.9%) |
| Migrants | 2,259 (2.0%) | 2,156 (1.6%) | 4,706 (2.1%) |
| **Urbanization (N, %)** |  |  |  |
| Urban | 83,376 (75.3%) | 83,147 (63.2%) | 128,746 (56.7%) |
| Semi-Urban | 14,953 (13.5%) | 23,026 (17.5%) | 42,221 (18.6%) |
| Rural | 12,380 (11.2%) | 25,457 (19.3%) | 56,216 (24.7%) |

^a^ Non-melanoma skin cancer (C44) was excluded.

^b^ Treatments were not mutually exclusive, so the percentages did not add up to 100%.

^c^ Over-all deaths and cancer specific deaths were followed up until the end of year 2020.

### Supplementary Table 2: Characteristics of the study population: newly diagnosed cancer cases during 2000-2019 by income

|  | **High income** | **Medium income** | **Low income** |
| --- | --- | --- | --- |
|  | **N = 169,928** | **N = 163,836** | **N = 135,758** |
| **Education (N, %)** |  |  |  |
| High | 76,751 (45.2%) | 23,659 (14.4%) | 10,299 (7.6%) |
| Medium | 41,672 (24.5%) | 51,944 (31.7%) | 38,014 (28.0%) |
| Low | 51,505 (30.3%) | 88,233 (53.9%) | 87,445 (64.4%) |
| **Gender (N, %)** |  |  |  |
| Men | 106,248 (62.5%) | 78,061 (47.6%) | 56,437 (41.6%) |
| Women | 63,680 (37.5%) | 85,775 (52.4%) | 79,321 (58.4%) |
| **Age at diagnosis (mean, SD)** | 68.39 (12.5) | 68.32 (12.6) | 67.72 (12.7) |
| **Source of diagnoses (N, %)** |  |  |  |
| Clinical | 162,627 (95.7%) | 154,100 (94.1%) | 125,595 (92.5%) |
| Death certificate only/Autopsy | 7,301 (4.3%) | 9,736 (5.9%) | 10,163 (7.5%) |
| **Cancer types (N, %)** ^a^ |  |  |  |
| Bladder | 3,878 (2.3%) | 3,353 (2.0%) | 2,230 (1.6%) |
| Breast | 25,321 (14.9%) | 29,951 (18.3%) | 24,042 (17.7%) |
| Colorectum | 17,096 (10.1%) | 16,238 (9.9%) | 13,735 (10.1%) |
| Lung | 11,672 (6.9%) | 16,027 (9.8%) | 14,185 (10.4%) |
| Melanoma | 9,239 (5.4%) | 6,230 (3.8%) | 4,207 (3.1%) |
| Prostate | 41,760 (24.6%) | 27,047 (16.5%) | 16,920 (12.5%) |
| Uteri | 3,707 (2.2%) | 5,188 (3.2%) | 5,466 (4.0%) |
| Others | 57,255 (33.7%) | 59,802 (36.5%) | 54,973 (40.5%) |
| **Cancer stage (N, %)** |  |  |  |
| Stage I-II | 47,426 (27.9%) | 46,442 (28.3%) | 39,366 (29.0%) |
| Stage III | 17,251 (10.2%) | 18,393 (11.2%) | 15,148 (11.2%) |
| Stage IV | 44,792 (26.4%) | 49,286 (30.1%) | 46,758 (34.4%) |
| Missing | 60,459 (35.6%) | 49,715 (30.3%) | 34,486 (25.4%) |
| **Treatment (N, %)** ^b^ |  |  |  |
| Surgery | 72,032 (42.4%) | 69,729 (42.6%) | 56,885 (41.9%) |
| Radiation | 39,305 (23.1%) | 30,210 (18.4%) | 17,966 (13.2%) |
| Chemotherapy | 36,273 (21.3%) | 32,206 (19.7%) | 22,049 (16.2%) |
| Hormone therapy | 36,604 (21.5%) | 34,561 (21.1%) | 25,075 (18.5%) |
| Other therapies | 8,446 (5.0%) | 7,469 (4.6%) | 5,168 (3.8%) |
| **Outcome (N, %)** ^c^ |  |  |  |
| Over-all deaths | 78,385 (46.1%) | 92,628 (56.5%) | 90,755 (66.9%) |
| Cancer specific deaths | 53,545 (31.5%) | 64,036 (39.1%) | 62,204 (45.8%) |
| **Survival years (mean, SD)** ^c^ | 4.79 (4.7) | 5.11 (5.3) | 5.31 (5.7) |
| **Marital status (N, %)** |  |  |  |
| Married | 103,892 (61.1%) | 84,138 (51.4%) | 70,380 (51.8%) |
| Unmarried | 15,938 (9.4%) | 21,778 (13.3%) | 24,299 (17.9%) |
| Divorced | 22,186 (13.1%) | 28,805 (17.6%) | 20,425 (15.0%) |
| Widowed | 27,912 (16.4%) | 29,115 (17.8%) | 20,654 (15.2%) |
| **Origin (N, %)** |  |  |  |
| Natives | 168,049 (98.9%) | 161,469 (98.6%) | 130,883 (96.4%) |
| Migrants | 1,879 (1.1%) | 2,367 (1.4%) | 4,875 (3.6%) |
| **Urbanization (N, %)** |  |  |  |
| Urban | 121,822 (71.7%) | 103,485 (63.2%) | 69,962 (51.5%) |
| Semi-Urban | 24,812 (14.6%) | 29,003 (17.7%) | 26,385 (19.4%) |
| Rural | 23,294 (13.7%) | 31,348 (19.1%) | 39,411 (29.0%) |

^a^ Non-melanoma skin cancer (C44) was excluded.

^b^ Treatments are not mutually exclusive, so the percentages do not add up to 100%.

^c^ Over-all deaths and cancer specific deaths were followed up until the end of year 2020.

### Supplementary Table 3: Age standardized cancer incidence rates (ASRs) and standardized rate ratios (SRRs) by cancer type and by stage across education groups, stratified by sex

| Cancer type | Cancer stage | High  ASR (95% CI) | Medium  ASR (95% CI) | Medium vs High  SSR (95% CI) | Low  ASR (95% CI) | Low vs High  SSR (95% CI) |
| --- | --- | --- | --- | --- | --- | --- |
| **Men** |  |  |  |  |  |  |
| All ^a^ | Total ^b^ | 679 (673–685) | 694 (688–700) | 1.02 (1.01–1.04) | 797 (792–801) | 1.17 (1.16–1.19) |
|  | Stage I-II | 189 (186––192) | 170 (168–173) | 0.90 (0.88–0.92) | 210 (207–212) | 1.11 (1.09–1.13) |
|  | Stage III-IV | 226 (222–229) | 253 (250–257) | 1.12 (1.10–1.15) | 323 (320–326) | 1.43 (1.41–1.46) |
| Prostate | Total ^b^ | 273 (269–276) | 246 (243–250) | 0.90 (0.89–0.92) | 262 (260–265) | 0.96 (0.94–0.98) |
|  | Stage I-II | 111 (108–113) | 91 (88–93) | 0.82 (0.79–0.85) | 107 (105–108) | 0.96 (0.94–0.99) |
|  | Stage III-IV | 40 (39–42) | 41 (39–42) | 1.02 (0.96–1.07) | 51 (49–52) | 1.26 (1.21–1.31) |
| Lung | Total ^b^ | 43 (42–45) | 76 (74–78) | 1.74 (1.66–1.82) | 117 (115–119) | 2.69 (2.58–2.79) |
|  | Stage I-II | 4 (4–5) | 7 (6–7) | 1.61 (1.39–1.87) | 12 (11–13) | 2.91 (2.57–3.30) |
|  | Stage III-IV | 27 (25–28) | 46 (45–48) | 1.74 (1.64–1.84) | 74 (73–75) | 2.79 (2.65–2.93) |
| Colorectum | Total ^b^ | 72 (70–74) | 72 (70–74) | 0.99 (0.96–1.03) | 78 (77–80) | 1.08 (1.05–1.12) |
|  | Stage I-II | 12 (11–13) | 11 (11–12) | 0.93 (0.84–1.02) | 15 (14–15) | 1.20 (1.11–1.30) |
|  | Stage III-IV | 40 (39–42) | 39 (38–41) | 0.98 (0.93–1.03) | 44 (43–45) | 1.10 (1.06–1.15) |
| Bladder | Total ^b^ | 25 (24–26) | 27 (26–29) | 1.10 (1.03–1.18) | 34 (33–35) | 1.38 (1.30–1.46) |
|  | Stage I-II | 12 (11–13) | 14 (13–15) | 1.13 (1.03–1.24) | 18 (17–19) | 1.48 (1.37–1.60) |
|  | Stage III-IV | 3 (2–3) | 3 (3–4) | 1.16 (0.95–1.41) | 4 (4–5) | 1.57 (1.33–1.85) |
| Melanoma | Total ^b^ | 42 (41–44) | 30 (28–31) | 0.70 (0.66–0.74) | 25 (24–26) | 0.60 (0.57–0.63) |
|  | Stage I-II | 11 (11–12) | 8 (8–9) | 0.73 (0.66–0.81) | 8 (7–8) | 0.68 (0.62–0.74) |
|  | Stage III-IV | 6 (6–7) | 5 (4–5) | 0.75 (0.66–0.86) | 5 (5–6) | 0.81 (0.72–0.91) |
| Others | Total ^b^ | 223 (220–227) | 243 (240–247) | 1.09 (1.07–1.11) | 280 (277–283) | 1.25 (1.23–1.28) |
|  | Stage I-II | 39 (38–40) | 40 (39–41) | 1.02 (0.98–1.08) | 51 (49–52) | 1.31 (1.25–1.36) |
|  | Stage III-IV | 109 (107–112) | 119 (117–121) | 1.09 (1.05–1.12) | 145 (143–147) | 1.32 (1.29–1.36) |
| **Women** |  |  |  |  |  |  |
| All ^a^ | Total ^b^ | 565 (560–570) | 548 (544–553) | 0.97 (0.96–0.98) | 593 (588–598) | 1.05 (1.04–1.06) |
|  | Stage I-II | 186 (183–189) | 168 (166–170) | 0.90 (0.88–0.92) | 182 (179–184) | 0.98 (0.96–1.00) |
|  | Stage III-IV | 232 (228–235) | 236 (233–238) | 1.02 (1.00–1.04) | 273 (270–277) | 1.18 (1.16–1.20) |
| Breast | Total ^b^ | 239 (235–242) | 199 (197–202) | 0.83 (0.82–0.85) | 193 (191–196) | 0.81 (0.80–0.83) |
|  | Stage I-II | 116 (114–118) | 92 (91–94) | 0.80 (0.78–0.82) | 91 (89–93) | 0.78 (0.76–0.80) |
|  | Stage III-IV | 93 (91–95) | 81 (79–83) | 0.87 (0.84–0.89) | 83 (81–85) | 0.89 (0.86–0.92) |
| Colorectum | Total ^b^ | 51 (49–52) | 53 (52–55) | 1.05 (1.01–1.09) | 56 (55–58) | 1.11 (1.07–1.16) |
|  | Stage I-II | 8 (8–9) | 8 (8–9) | 1.04 (0.94–1.15) | 12 (11–12) | 1.41 (1.28–1.54) |
|  | Stage III-IV | 29 (28–30) | 30 (29–31) | 1.03 (0.98–1.09) | 32 (31–33) | 1.09 (1.04–1.15) |
| Lung | Total ^b^ | 21 (20–22) | 30 (29–31) | 1.39 (1.31–1.47) | 44 (42–45) | 2.04 (1.93–2.16) |
|  | Stage I-II | 2 (2–2) | 3 (3–3) | 1.55 (1.28–1.88) | 5 (4–5) | 2.45 (2.05–2.93) |
|  | Stage III-IV | 13 (12–14) | 17 (17–18) | 1.33 (1.23–1.43) | 27 (26–28) | 2.08 (1.94–2.23) |
| Melanoma | Total ^b^ | 31 (30–32) | 24 (23–25) | 0.77 (0.73–0.81) | 19 (18–20) | 0.62 (0.58–0.66) |
|  | Stage I-II | 9 (9–10) | 7 (7–8) | 0.81 (0.74–0.89) | 7 (6–7) | 0.73 (0.66–0.81) |
|  | Stage III-IV | 3 (3–3) | 3 (2–3) | 0.83 (0.70–0.98) | 3 (2–3) | 0.83 (0.69–0.98) |
| Corpus uteri | Total ^b^ | 36 (35–38) | 35 (34–36) | 0.96 (0.92–1.01) | 38 (37–39) | 1.04 (1.00–1.09) |
|  | Stage I-II | 16 (15–17) | 16 (16–17) | 1.03 (0.96–1.10) | 19 (18–20) | 1.22 (1.14–1.31) |
|  | Stage III-IV | 6 (5–6) | 6 (6–7) | 1.08 (0.96–1.21) | 7 (7–8) | 1.27 (1.13–1.42) |
| Others | Total ^b^ | 187 (184–190) | 207 (205–210) | 1.11 (1.09–1.13) | 242 (239–245) | 1.30 (1.27–1.32) |
|  | Stage I-II | 35 (34–36) | 40 (39–42) | 1.15 (1.10–1.21) | 49 (47–50) | 1.39 (1.33–1.46) |
|  | Stage III-IV | 87 (85–90) | 99 (97–100) | 1.13 (1.09–1.16) | 122 (120–124) | 1.39 (1.35–1.43) |

^a^ Non-melanoma skin cancer (C44) was excluded.

^b^ Cancer cases with missing stage information were included.

### Supplementary Table 4: Age standardized cancer incidence rates (ASRs) and standardized rate ratios (SRRs) by cancer type and by stage across income groups, stratified by sex

| Cancer type | Cancer stage | High  ASR (95% CI) | Medium  ASR (95% CI) | Medium vs High  SSR (95% CI) | Low  ASR (95% CI) | Low vs High  SSR (95% CI) |
| --- | --- | --- | --- | --- | --- | --- |
| **Men** |  |  |  |  |  |  |
| All ^a^ | Total ^b^ | 679 (675–683) | 764 (759–770) | 1.13 (1.12–1.14) | 849 (841–856) | 1.25 (1.24–1.26) |
|  | Stage I-II | 179 (177–181) | 202 (199–205) | 1.13 (1.11–1.15) | 226 (222–230) | 1.26 (1.24–1.29) |
|  | Stage III-IV | 230 (228–233) | 297 (293–300) | 1.29 (1.27–1.31) | 366 (361–371) | 1.59 (1.56–1.62) |
| Prostate | Total ^b^ | 264 (261–266) | 260 (257–264) | 0.99 (0.97–1.00) | 268 (264–273) | 1.02 (1.00–1.04) |
|  | Stage I-II | 102 (100–103) | 106 (104–108) | 1.04 (1.01–1.06) | 110 (108–113) | 1.08 (1.05–1.12) |
|  | Stage III-IV | 41 (40–42) | 48 (46–49) | 1.18 (1.14–1.22) | 59 (57–61) | 1.45 (1.39–1.51) |
| Lung | Total ^b^ | 55 (54–56) | 102 (100–104) | 1.86 (1.81–1.92) | 138 (135–141) | 2.51 (2.43–2.59) |
|  | Stage I-II | 5 (5–5) | 10 (9–11) | 2.06 (1.88–2.27) | 15 (14–16) | 3.06 (2.78–3.37) |
|  | Stage III-IV | 33 (32–34) | 63 (61–64) | 1.91 (1.84–1.98) | 89 (87–91) | 2.70 (2.60–2.80) |
| Colorectum | Total ^b^ | 71 (70–73) | 77 (75–78) | 1.08 (1.04–1.11) | 81 (78–83) | 1.13 (1.10–1.17) |
|  | Stage I-II | 12 (11–12) | 14 (13–15) | 1.21 (1.13–1.30) | 16 (15–17) | 1.37 (1.27–1.48) |
|  | Stage III-IV | 40 (39–41) | 43 (42–45) | 1.09 (1.05–1.13) | 46 (44–48) | 1.16 (1.11–1.21) |
| Bladder | Total ^b^ | 25 (25–26) | 33 (32–35) | 1.32 (1.26–1.39) | 36 (34–37) | 1.41 (1.33–1.49) |
|  | Stage I-II | 12 (12–13) | 18 (17–19) | 1.46 (1.37–1.56) | 20 (19–21) | 1.63 (1.52–1.76) |
|  | Stage III-IV | 3 (3–3) | 4 (4–4) | 1.43 (1.25–1.64) | 5 (5–6) | 1.91 (1.65–2.21) |
| Melanoma | Total ^b^ | 37 (37–38) | 27 (26–28) | 0.73 (0.70–0.76) | 23 (22–24) | 0.61 (0.57–0.64) |
|  | Stage I-II | 10 (9–10) | 8 (8–9) | 0.85 (0.78–0.93) | 8 (7–8) | 0.78 (0.70–0.86) |
|  | Stage III-IV | 6 (5–6) | 5 (5–6) | 0.96 (0.86–1.07) | 5 (5–6) | 0.96 (0.85–1.09) |
| Others | Total ^b^ | 226 (224–229) | 264 (261–267) | 1.17 (1.15–1.19) | 303 (299–308) | 1.34 (1.32–1.36) |
|  | Stage I-II | 38 (37–39) | 46 (45–47) | 1.20 (1.16–1.25) | 57 (55–59) | 1.49 (1.43–1.55) |
|  | Stage III-IV | 109 (107–111) | 133 (131–136) | 1.22 (1.20–1.25) | 162 (159–165) | 1.49 (1.45–1.52) |
| **Women** |  |  |  |  |  |  |
| All ^a^ | Total ^b^ | 554 (550–558) | 565 (562–569) | 1.02 (1.01–1.03) | 595 (591–599) | 1.07 (1.06–1.09) |
|  | Stage I-II | 172 (169–174) | 175 (173–177) | 1.02 (1.00–1.04) | 189 (187–192) | 1.10 (1.08–1.12) |
|  | Stage III-IV | 226 (223–229) | 246 (243–248) | 1.09 (1.07–1.10) | 276 (273–279) | 1.22 (1.20–1.24) |
| Breast | Total ^b^ | 227 (225–230) | 205 (203–207) | 0.90 (0.89–0.92) | 193 (191–196) | 0.85 (0.83–0.86) |
|  | Stage I-II | 108 (106–110) | 97 (95–98) | 0.90 (0.87–0.92) | 90 (89–92) | 0.84 (0.81–0.86) |
|  | Stage III-IV | 89 (88–91) | 84 (83–86) | 0.94 (0.92–0.97) | 82 (81–84) | 0.92 (0.89–0.95) |
| Colorectum | Total ^b^ | 50 (49–51) | 54 (52–55) | 1.07 (1.03–1.11) | 59 (58–60) | 1.18 (1.14–1.22) |
|  | Stage I-II | 8 (7–8) | 9 (9–10) | 1.22 (1.12–1.33) | 13 (12–13) | 1.66 (1.53–1.80) |
|  | Stage III-IV | 28 (27–29) | 30 (29–31) | 1.07 (1.02–1.12) | 34 (33–35) | 1.20 (1.15–1.26) |
| Lung | Total ^b^ | 26 (25–27) | 35 (34–36) | 1.33 (1.27–1.39) | 36 (35–37) | 1.36 (1.30–1.42) |
|  | Stage I-II | 2 (2–3) | 4 (3–4) | 1.52 (1.31–1.76) | 4 (4–4) | 1.71 (1.48–1.98) |
|  | Stage III-IV | 15 (14–16) | 21 (20–22) | 1.39 (1.31–1.47) | 23 (22–24) | 1.52 (1.43–1.61) |
| Melanoma | Total ^b^ | 31 (30–32) | 24 (23–25) | 0.78 (0.74–0.82) | 21 (20–21) | 0.66 (0.63–0.70) |
|  | Stage I-II | 9 (8–9) | 8 (7–8) | 0.87 (0.80–0.96) | 8 (7–8) | 0.88 (0.80–0.96) |
|  | Stage III-IV | 3 (2–3) | 3 (2–3) | 1.00 (0.86–1.17) | 3 (3–3) | 1.14 (0.98–1.34) |
| Corpus uteri | Total b | 32 (31–33) | 34 (33–35) | 1.06 (1.02–1.11) | 41 (40–42) | 1.28 (1.23–1.34) |
|  | Stage I-II | 13 (12–14) | 16 (15–17) | 1.24 (1.17–1.33) | 22 (21–23) | 1.71 (1.60–1.82) |
|  | Stage III-IV | 5 (5–6) | 6 (6–6) | 1.15 (1.03–1.27) | 8 (7–8) | 1.53 (1.38–1.69) |
| Others | Total ^b^ | 187 (185–190) | 214 (211–216) | 1.14 (1.12–1.16) | 246 (243–248) | 1.31 (1.29–1.34) |
|  | Stage I-II | 32 (31–33) | 42 (40–43) | 1.29 (1.24–1.35) | 53 (51–54) | 1.64 (1.57–1.71) |
|  | Stage III-IV | 86 (84–88) | 102 (101–104) | 1.19 (1.16–1.22) | 126 (125–128) | 1.47 (1.44–1.51) |

^a^ Non-melanoma skin cancer (C44) was excluded.

^b^ Cancer cases with missing stage information were included.

### Supplementary Table 5: Patterns of missing information on stage at diagnosis by education and income

|  |  |  | Education | Income |
| --- | --- | --- | --- | --- |
| Sex | Type | SES level | OR (95% CI) ^a^ | OR (95% CI) ^a^ |
| Men | All ^b^ | Low | 1.01 (1.00–1.03) | 1.08 (1.06–1.10) |
|  |  | Medium | 1.01 (0.99–1.02) | 1.01 (0.99–1.03) |
|  |  | High | Ref | Ref |
|  | Prostate | Low | 1.03 (1.01–1.06) | 1.13 (1.10–1.17) |
|  |  | Medium | 1.02 (1.00–1.05) | 1.03 (1.00–1.06) |
|  |  | High | Ref | Ref |
|  | Lung | Low | 0.97 (0.93–1.01) | 1.15 (1.09–1.21) |
|  |  | Medium | 0.94 (0.90–0.97) | 1.05 (0.98–1.12) |
|  |  | High | Ref | Ref |
|  | Colorectum | Low | 0.97 (0.93–1.01) | 1.14 (1.07–1.21) |
|  |  | Medium | 1.04 (0.99–1.09) | 1.00 (0.94–1.07) |
|  |  | High | Ref | Ref |
|  | Bladder | Low | 1.04 (0.97–1.11) | 1.00 (0.92–1.09) |
|  |  | Medium | 0.93 (0.88–0.99) | 0.97 (0.89–1.06) |
|  |  | High | Ref | Ref |
|  | Melanoma | Low | 0.90 (0.84–0.97) | 0.82 (0.76–0.88) |
|  |  | Medium | 0.97 (0.90–1.05) | 0.89 (0.82–0.97) |
|  |  | High | Ref | Ref |
|  | Others | Low | 1.02 (1.00–1.05) | 1.01 (0.98–1.05) |
|  |  | Medium | 1.01 (0.99–1.04) | 1.02 (0.98–1.05) |
|  |  | High | Ref | Ref |
| Women | All ^b^ | Low | 1.01 (0.99–1.02) | 1.05 (1.03–1.07) |
|  |  | Medium | 1.01 (0.99–1.03) | 1.02 (1.00–1.04) |
|  |  | High | Ref | Ref |
|  | Breast | Low | 1.06 (1.02–1.09) | 1.13 (1.08–1.17) |
|  |  | Medium | 1.04 (1.00–1.08) | 1.01 (0.96–1.05) |
|  |  | High | Ref | Ref |
|  | Lung | Low | 1.13 (1.07–1.20) | 1.07 (1.00–1.15) |
|  |  | Medium | 1.18 (1.12–1.25) | 1.07 (0.99–1.16) |
|  |  | High | Ref | Ref |
|  | Colorectum | Low | 1.04 (1.00–1.09) | 1.03 (0.98–1.08) |
|  |  | Medium | 1.08 (1.04–1.13) | 1.02 (0.96–1.08) |
|  |  | High | Ref | Ref |
|  | Corpus uteri | Low | 0.93 (0.88–0.98) | 1.05 (0.99–1.11) |
|  |  | Medium | 0.97 (0.92–1.02) | 1.03 (0.97–1.11) |
|  |  | High | Ref | Ref |
|  | Melanoma | Low | 0.96 (0.90–1.01) | 0.89 (0.82–0.97) |
|  |  | Medium | 1.00 (0.93–1.07) | 1.01 (0.92–1.10) |
|  |  | High | Ref | Ref |
|  | Others | Low | 1.00 (0.97–1.02) | 1.05 (1.03–1.08) |
|  |  | Medium | 0.98 (0.95–1.00) | 1.02 (0.99–1.05) |
|  |  | High | Ref | Ref |

^a^ the outcome is missing status of stage at diagnosis (missing or non-missing). The mean OR and 95% CI of low or medium education (income) comparing with high education (income) estimated from logistic regression models, adjusted with cancer type, age at cancer diagnosed (linear, square and quadratic terms), year at diagnosis, year of birth, marital status, origin, region of residence (totally 19 regions) and urbanization of the region (urban, semi-urban and rural).

^b^ cancer type is further adjusted in the models.

### Supplementary Figure 1. Study design and flow diagram


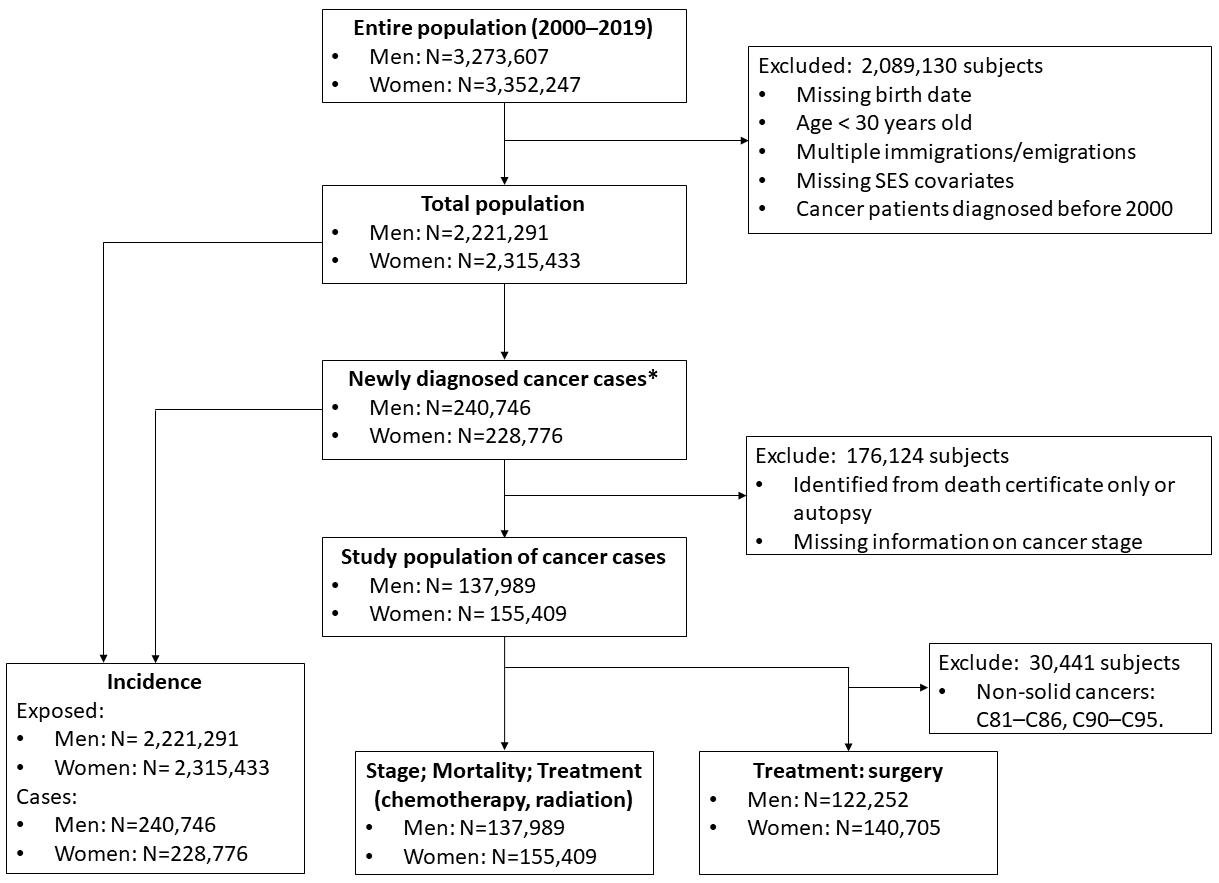


* Non-melanoma skin cancer (C44) was excluded.

Among the total target population (2,221,291 men and 2,315,433 women), a total of 240,746 men and 228,776 women were newly diagnosed with cancer between 2000 and 2019. For analyses on stage at diagnosis, chemotherapy, radiation therapy, and mortality, a total of 137,989 men and 155,409 women cancer cases were included, while cancer cases identified based on a death certificate or autopsy only and those with missing information on the stage at diagnosis were excluded. For the analysis of treatment with surgery, a total of 122,252 men and 140,705 women diagnosed with solid cancer were included, while those diagnosed with lymphoma (C81–C86), multiple myeloma (C90), and leukemia (C91–C95) were excluded.

Supplementary Figure 2. Standardized rate ratios (SRRs) of age standardized rates (ASRs) for different cancer types across income groups, stratified by sex
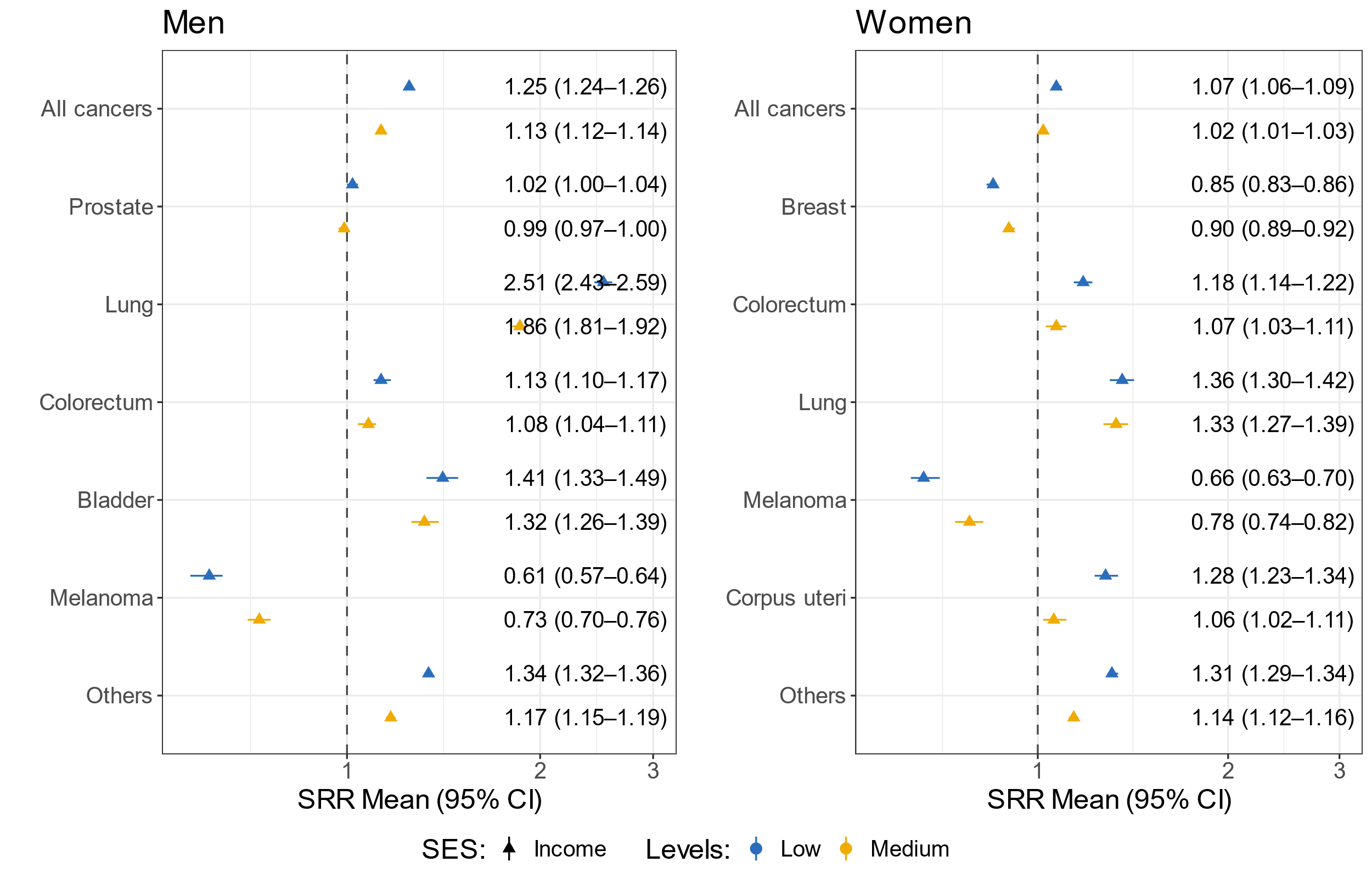


This figure shows the SRR between low (medium) income groups with high income groups. Dots with bar are the SRRs and corresponding 95% CI of cancer incidences (ASRs) in men (women) with medium (yellow) and low (blue) income level comparing with high income level.

### Supplementary Figure 3. Odds ratios (OR) of being diagnosed with stage IV cancer (ref. stage I-II-III cancer) across income groups by cancer type, stratified by sex


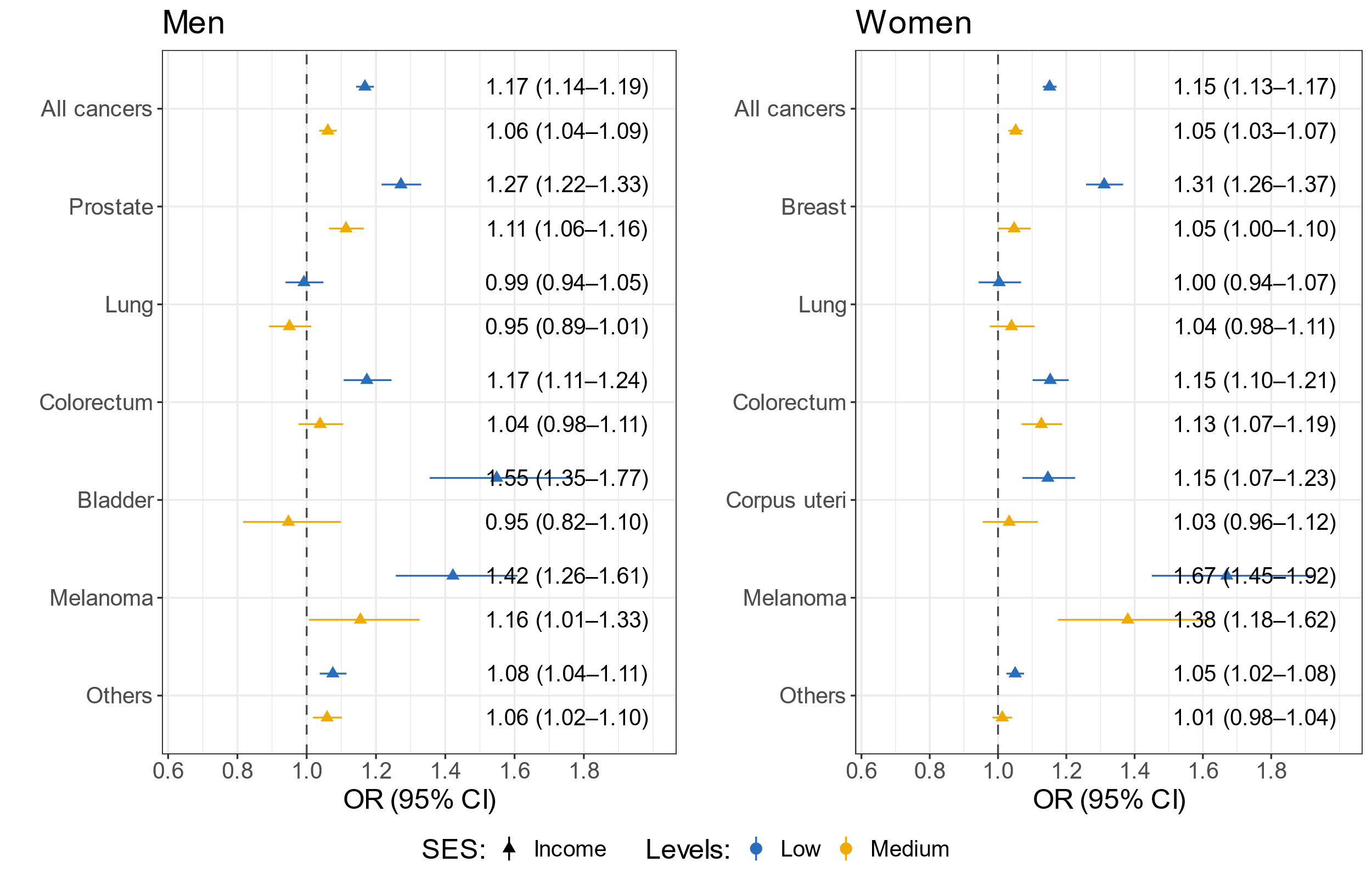


The models are adjusted for age at cancer diagnosis, year at diagnosis, year of birth, marital status, origin, region of residence, urbanization of the region, and income level stratified by cancer type. For all-cancer models, cancer type is adjusted as a dummy factor. The dots with bars represent the odds ratios (OR) and corresponding 95% confidence intervals (95% CI) for being diagnosed with stage IV cancer among individuals with low (blue) and medium (yellow) income levels compared to those with high income levels.

### Supplementary Figure 4. Odds ratios (OR) of being treated with surgery (ref. no surgery) for solid cancers across income groups by cancer type, stratified by sex


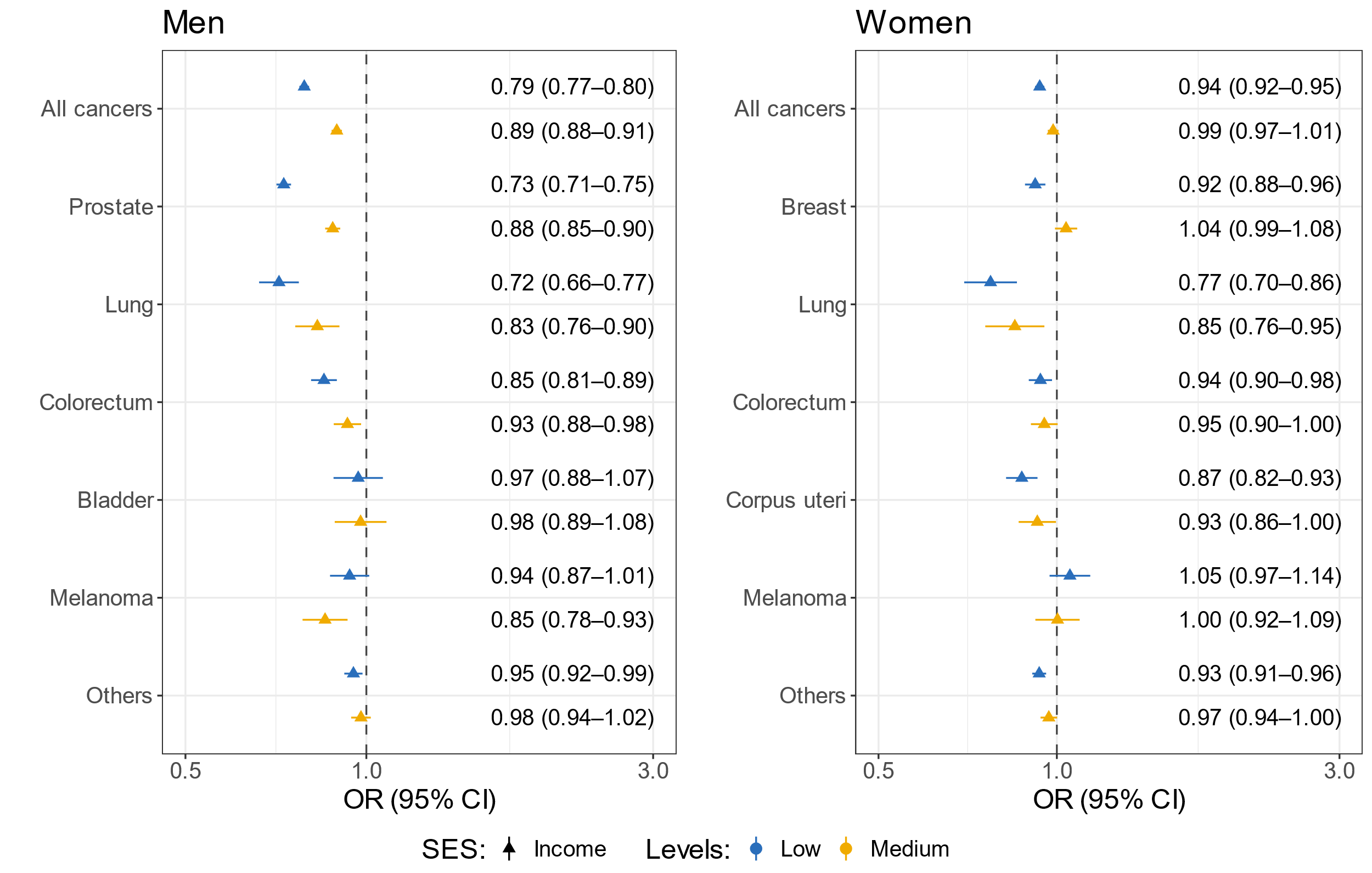


All solid cancer types were included, while lymphoma (C81–C86), multiple myeloma (C90), and leukemia (C91–C95) were excluded. The models are adjusted for stage at diagnosis, age at cancer diagnosis, year at diagnosis, year of birth, marital status, origin, region of residence, urbanization of the region, and income level stratified by cancer type. For prostate cancer, surgery and radiation therapy were combined together. For lung cancer, only non-small-cell lung cancer was included. For overall-cancer models, cancer type is adjusted as a dummy factor. The dots with bars represent the odds ratios (OR) and corresponding 95% confidence intervals (CI) for being treated with surgery among individuals with medium (yellow) and low (blue) income levels compared to those with high income levels.

### Supplementary Figure 5. Odds ratios (OR) of being treated with chemotherapy (ref. no chemotherapy) across education groups by cancer type, stratified by sex


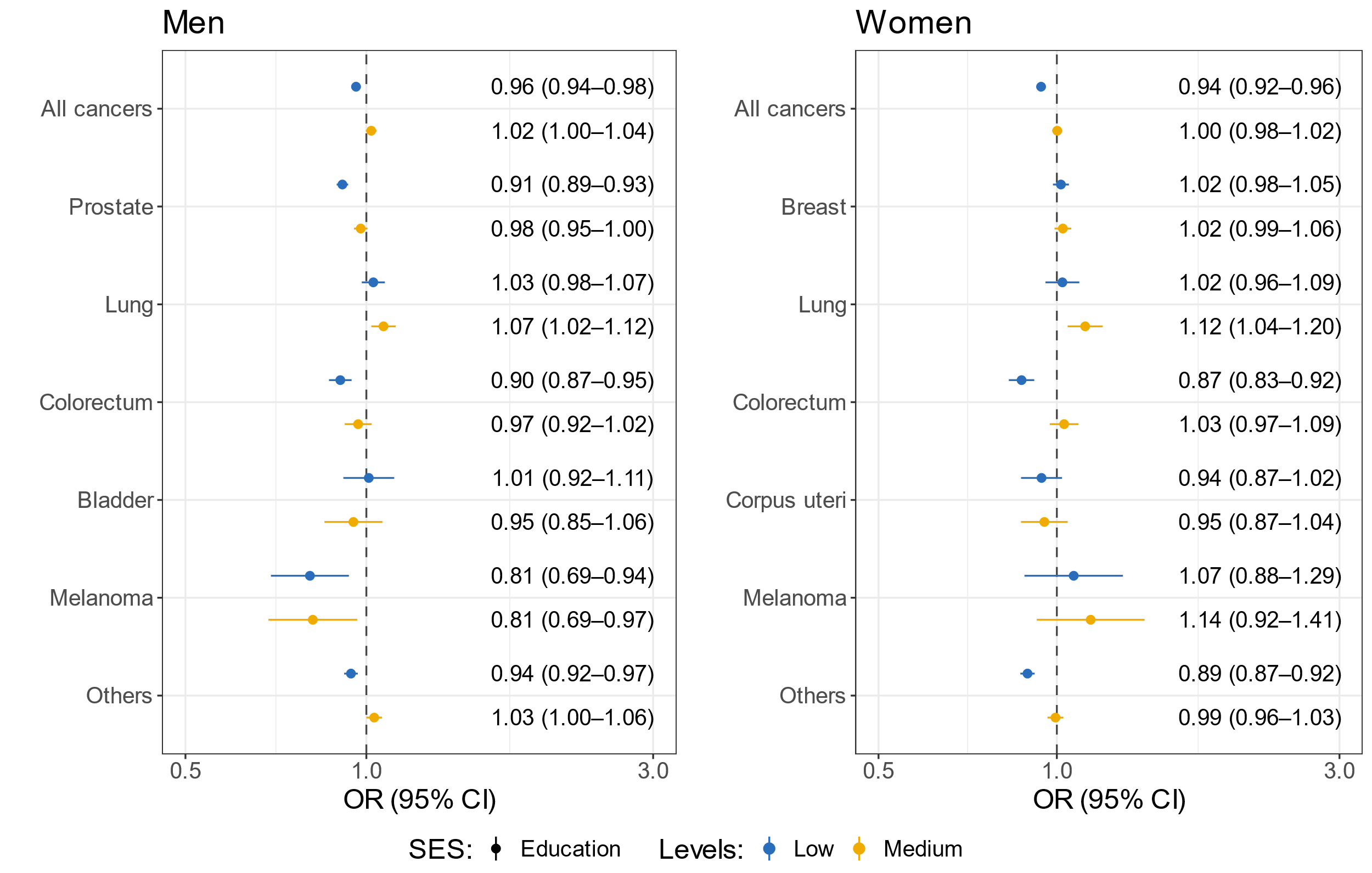


The models are adjusted for stage at cancer diagnosis, age at diagnosis, year at diagnosis, year of birth, marital status, origin, region of residence, urbanization of the region, and education level stratified by cancer type. For the all cancer models, cancer type is adjusted as a dummy factor. The dots with bars represent the odds ratios (OR) and corresponding 95% confidence intervals (CI) for being treated with surgery among individuals with medium (yellow) and low (blue) education levels compared to those with high education levels.

### Supplementary Figure 6. Odds ratios (OR) of being treated with chemotherapy (ref. no chemotherapy) across income groups by cancer type, stratified by sex


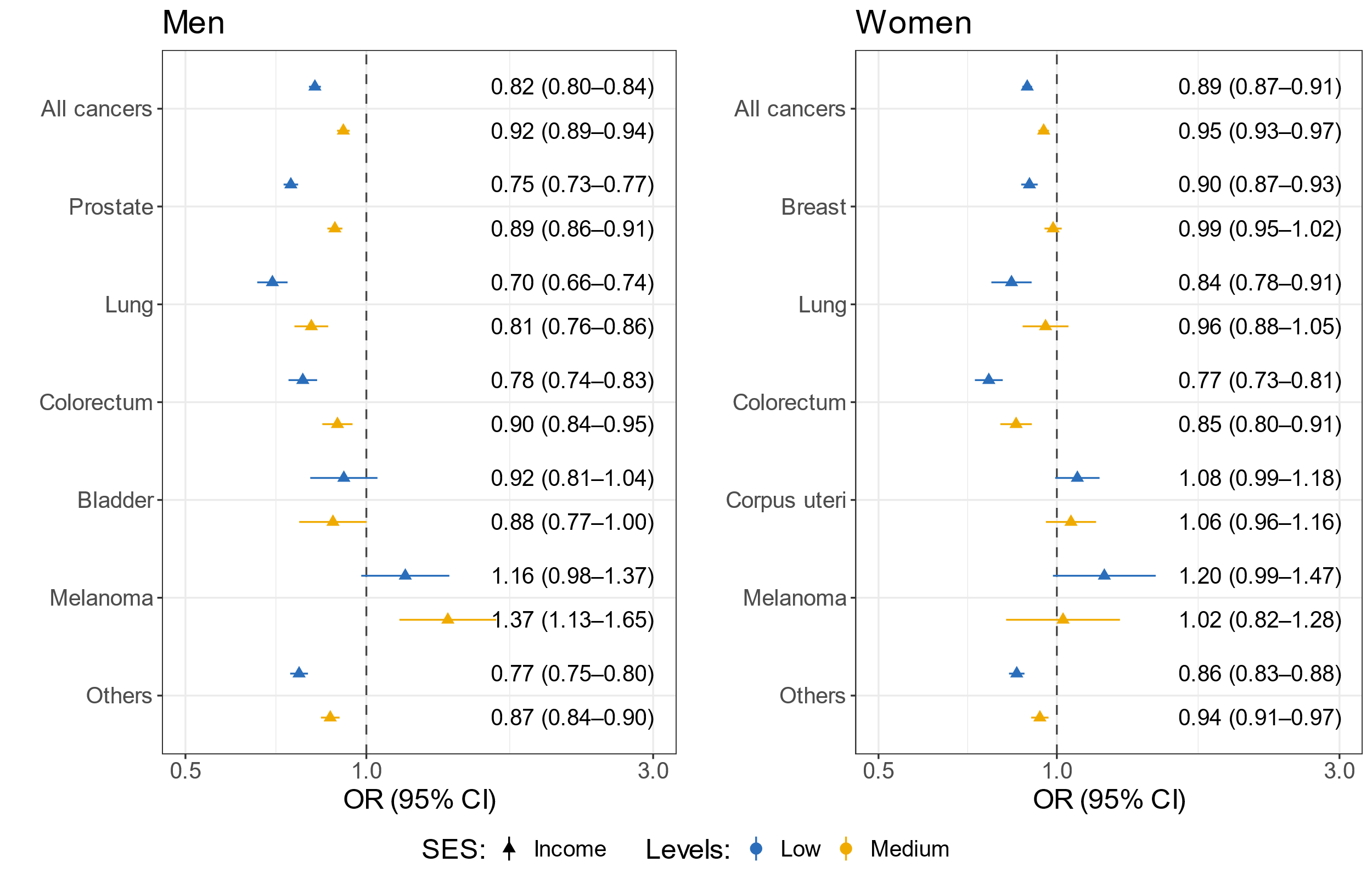


The models are adjusted for stage at diagnosis, age at cancer diagnosis, year at diagnosis, year of birth, marital status, origin, region of residence, urbanization of the region, and income level stratified by cancer type. For lung cancer, only non-small-cell lung cancer was included. For all-cancer models, cancer type is adjusted as a dummy factor. The dots with bars represent the odds ratios (OR) and corresponding 95% confidence intervals (CI) for being treated with surgery among individuals with medium (yellow) and low (blue) income levels compared to those with high income levels.

### Supplementary Figure 7. Odds ratios (OR) of being treated with radiation therapy (ref. no radiation therapy) across education groups by cancer type, stratified by sex


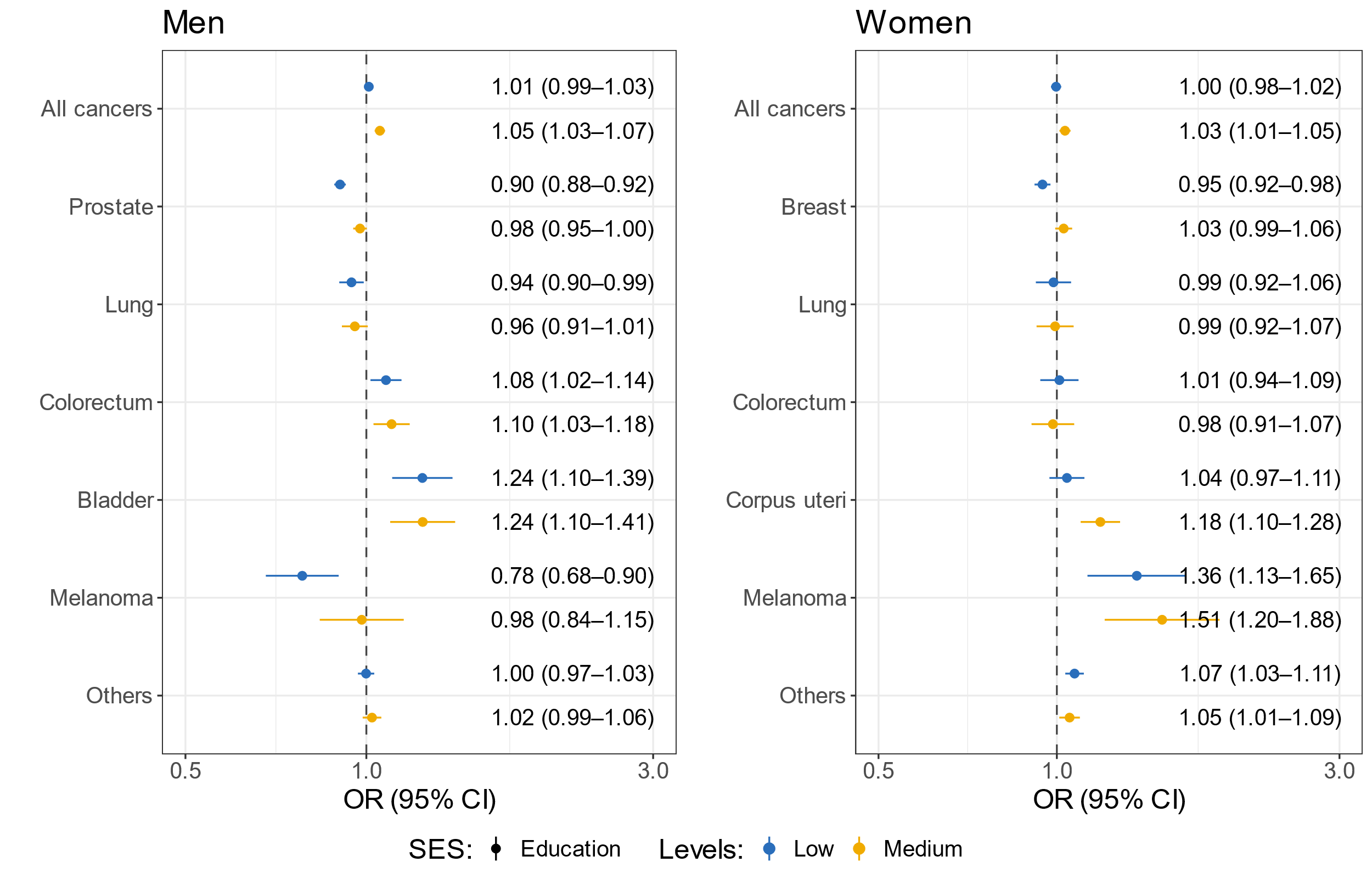


The models are adjusted for stage at cancer diagnosis, age at diagnosis, year at diagnosis, year of birth, marital status, origin, region of residence, urbanization of the region, and education level stratified by cancer type. For prostate cancer, surgery and radiation therapy are combined. For the all cancer models, cancer type is adjusted as a dummy factor. The dots with bars represent the odds ratios (OR) and corresponding 95% confidence intervals (CI) for being treated with surgery among individuals with medium (yellow) and low (blue) education levels compared to those with high education levels.

### Supplementary Figure 8. Odds ratios (OR) of being treated with radiation therapy (ref. no radiation therapy) across income groups by cancer type, stratified by sex


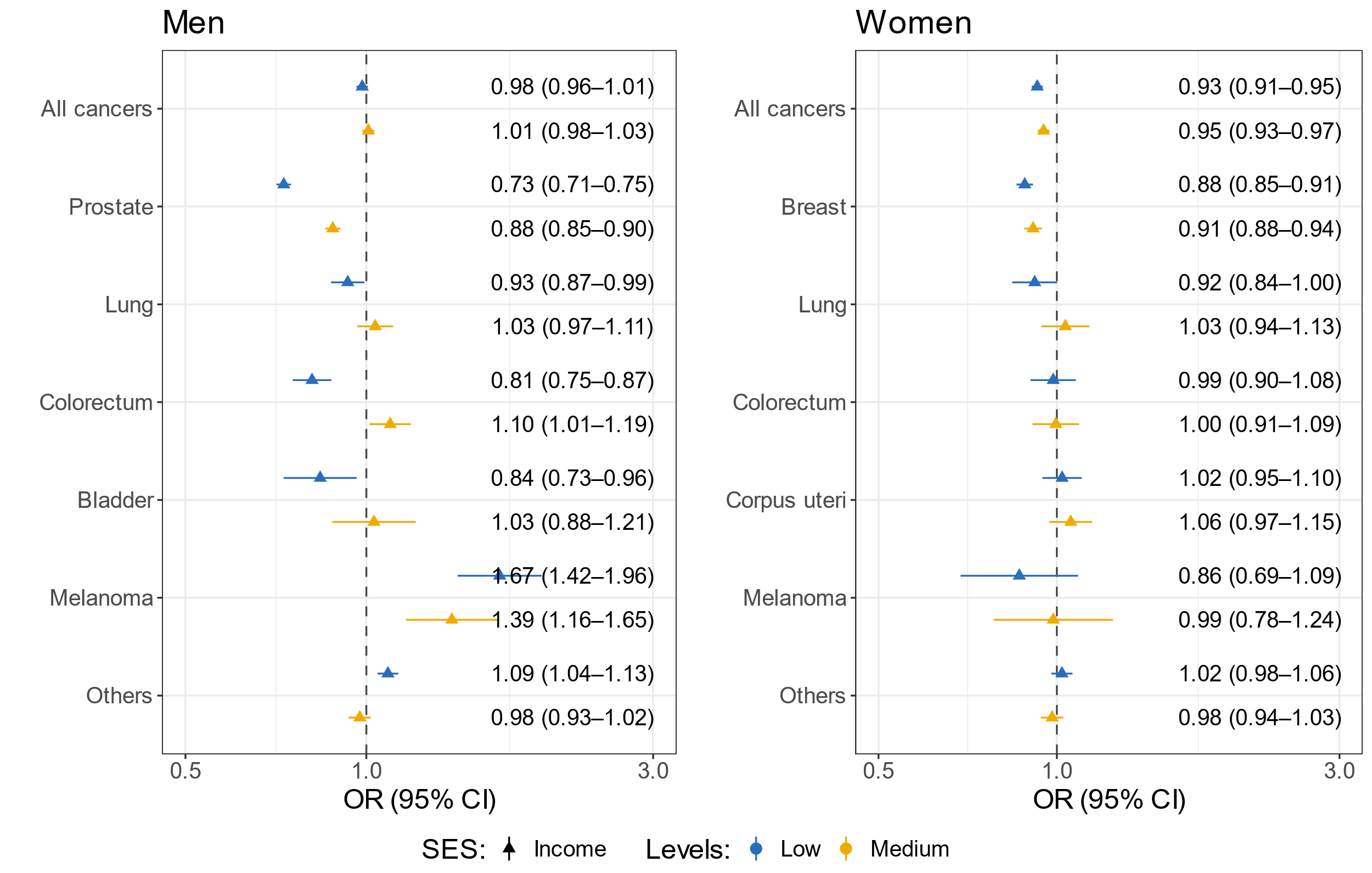


The models are adjusted for stage at diagnosis, age at cancer diagnosis, year at diagnosis, year of birth, marital status, origin, region of residence, urbanization of the region, and income level stratified by cancer type. For prostate cancer, surgery and radiation therapy were combined together. For lung cancer, only non-small-cell lung cancer was included. For overall-cancer models, cancer type is adjusted as a dummy factor. The dots with bars represent the odds ratios (OR) and corresponding 95% confidence intervals (CI) for being treated with surgery among individuals with medium (yellow) and low (blue) income levels compared to those with high income levels.

### Supplementary Figure 9. Hazard Ratio (HR) of cancer-specific mortality across income groups by cancer type, stratified by sex


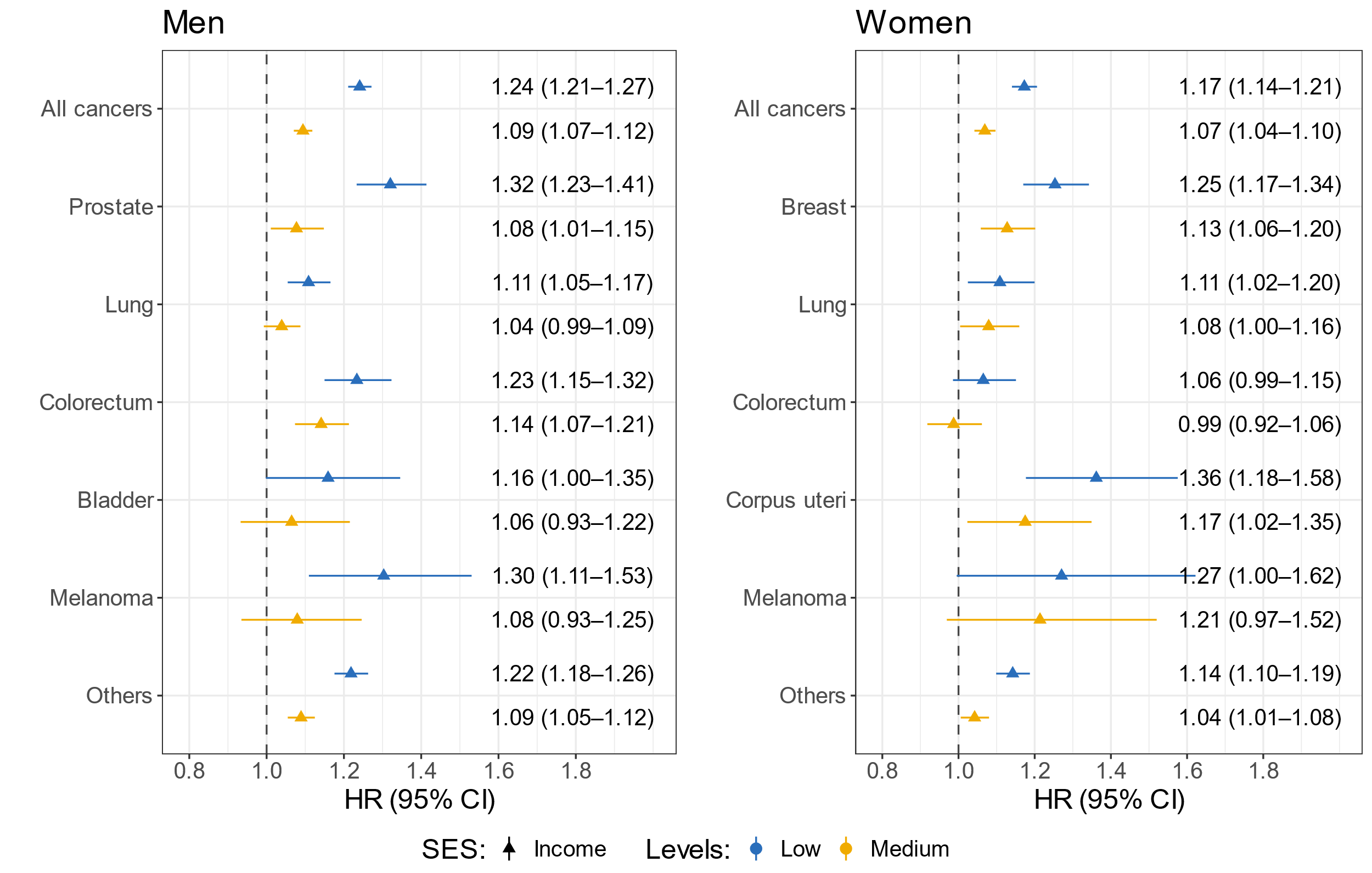


Non-melanoma skin cancer (C44) was excluded. The models are adjusted for stage at diagnosis, treatment, age at cancer diagnosis, year at diagnosis, year of birth, marital status, origin, region of residence, urbanization of the region, and income level stratified by cancer type. For overall-cancer models, cancer type is adjusted as a dummy factor. The dots with bars represent the hazard ratios (HR) and corresponding 95% confidence intervals (95% CI) for mortality among cancer patients with low (blue) medium (yellow) income levels compared to those with high income levels.

Supplementary Figure 10: Sensitivity analyses of SES factors on being diagnosed with stage IV cancer for all cancers, stratified by sex **
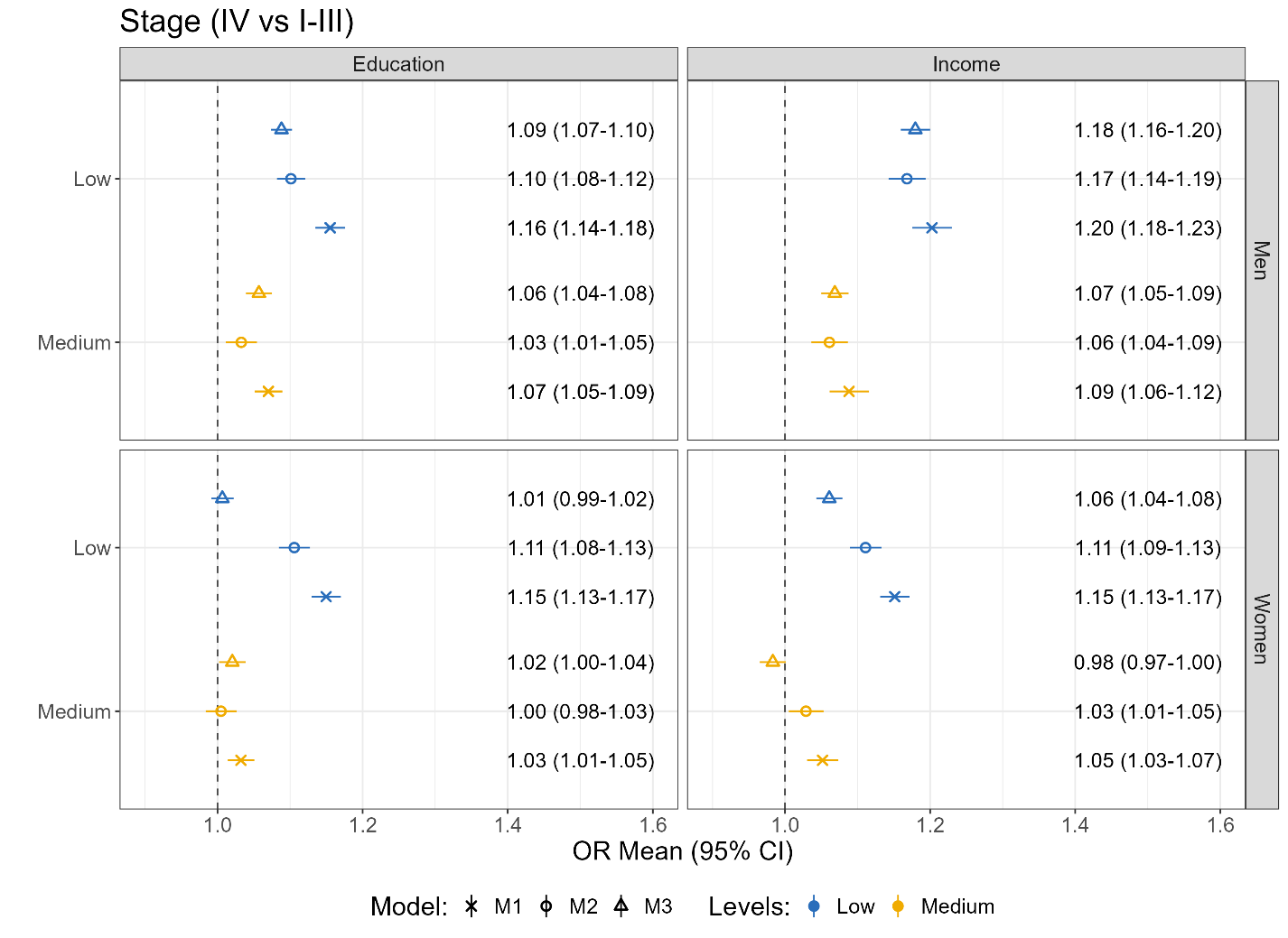
**

The outcome is diagnosed with earlier or later stage cancer. The figures show the mean OR and 95% CI of low or medium education (income) comparing with high education (income) estimated from logistic regression models. Covariates are adjusted in the models sequentially.

M1: logistic regression with education (or income), cancer type, age at cancer diagnosis, year at diagnosis, year of birth, marital status, origin, region of residence and urbanization of the region.

M2: M1 + income (or education)

M3: M2 + education * income

### Supplementary Figure 11: Sensitivity analyses of SES factors on treatment (surgery) for all cancers, stratified by sex

**
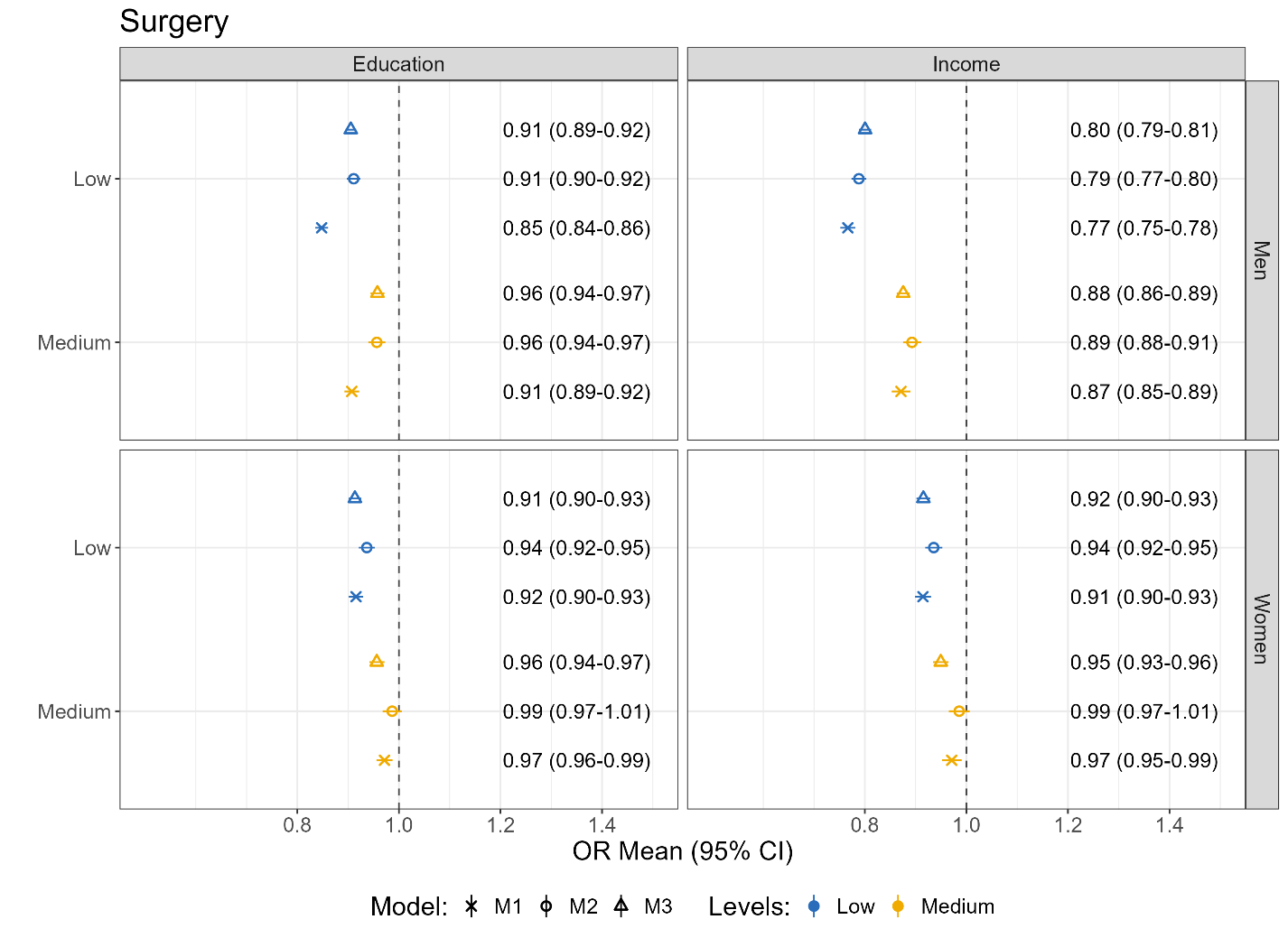
**

The outcome is taking surgery or not. The figures show the mean OR and 95% CI of low or medium education (income) comparing with high education (income) estimated from logistic regression models. Covariates were adjusted in the models sequentially. The upper panels are for samples diagnosed with early stage cancers. The lower panels are for sample diagnosed with later stage cancers.

M1: logistic regression with education (or income), cancer type, stage at diagnosis, age at cancer diagnosis, year at diagnosis, year of birth, marital status, origin, region of residence and urbanization of the region.

M2: M1 + income (or education)

M3: M2 + education * income

### Supplementary Figure 12: Sensitivity analyses of SES factors on treatment (chemotherapy) for all cancers, stratified by sex

**
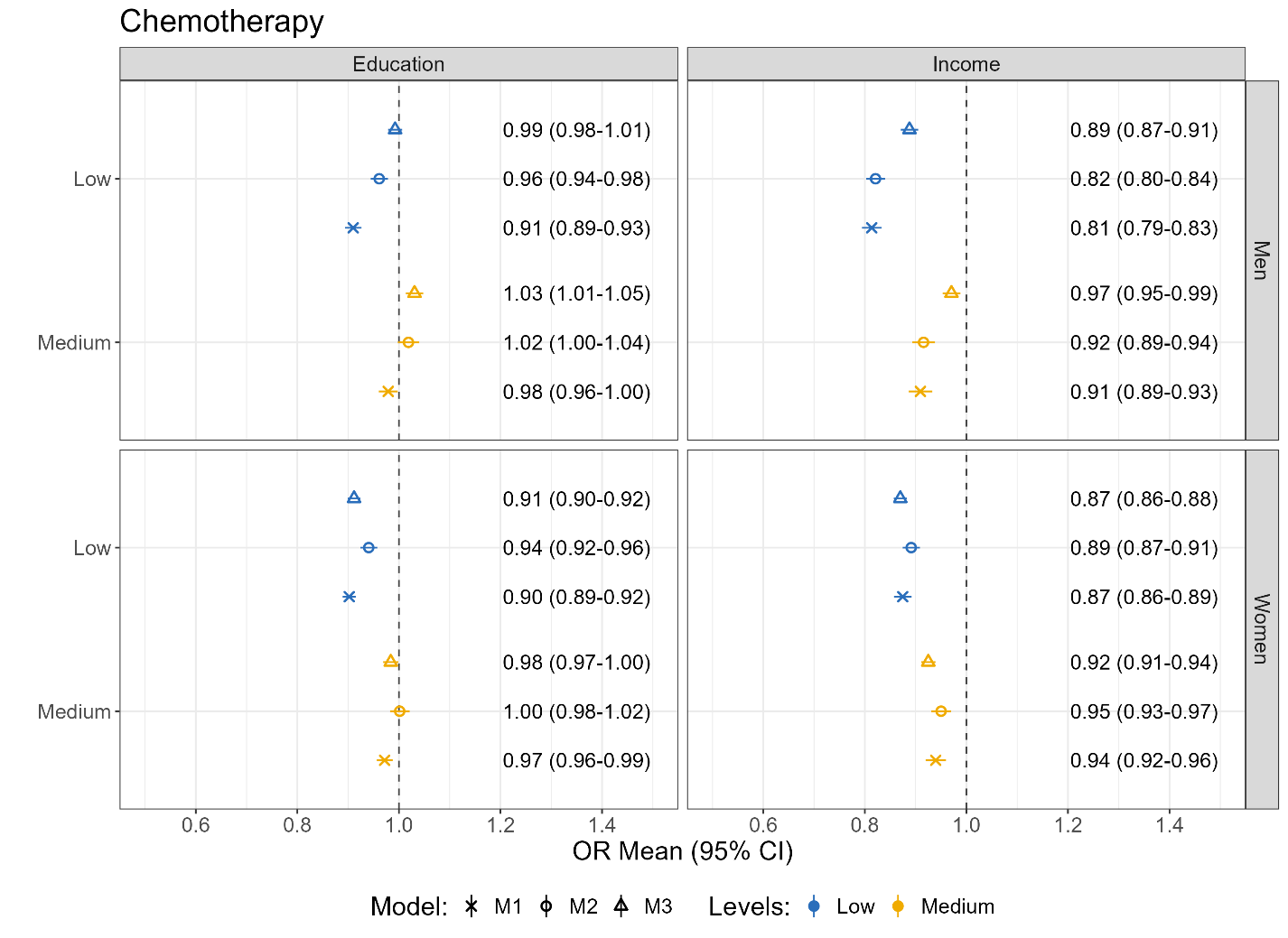
**

The outcome is taking chemo therapy or not. The figures show the mean OR and 95% CI of low or medium education (income) comparing with high education (income) estimated from logistic regression models. Covariates were adjusted in the models sequentially. The upper panels are for samples diagnosed with early stage cancers. The lower panels are for sample diagnosed with later stage cancers.

M1: logistic regression with education (or income), cancer type, stage at diagnosis, age at cancer diagnosis (linear, square and quadratic terms), year at diagnosis, year of birth, marital status, origin, region of residence and urbanization of the region.

M2: M1 + income (or education)

M3: M2 + education * income

### Supplementary Figure 13: Sensitivity analyses of SES factors on treatment (radiation therapy) for all cancers, stratified by sex

**
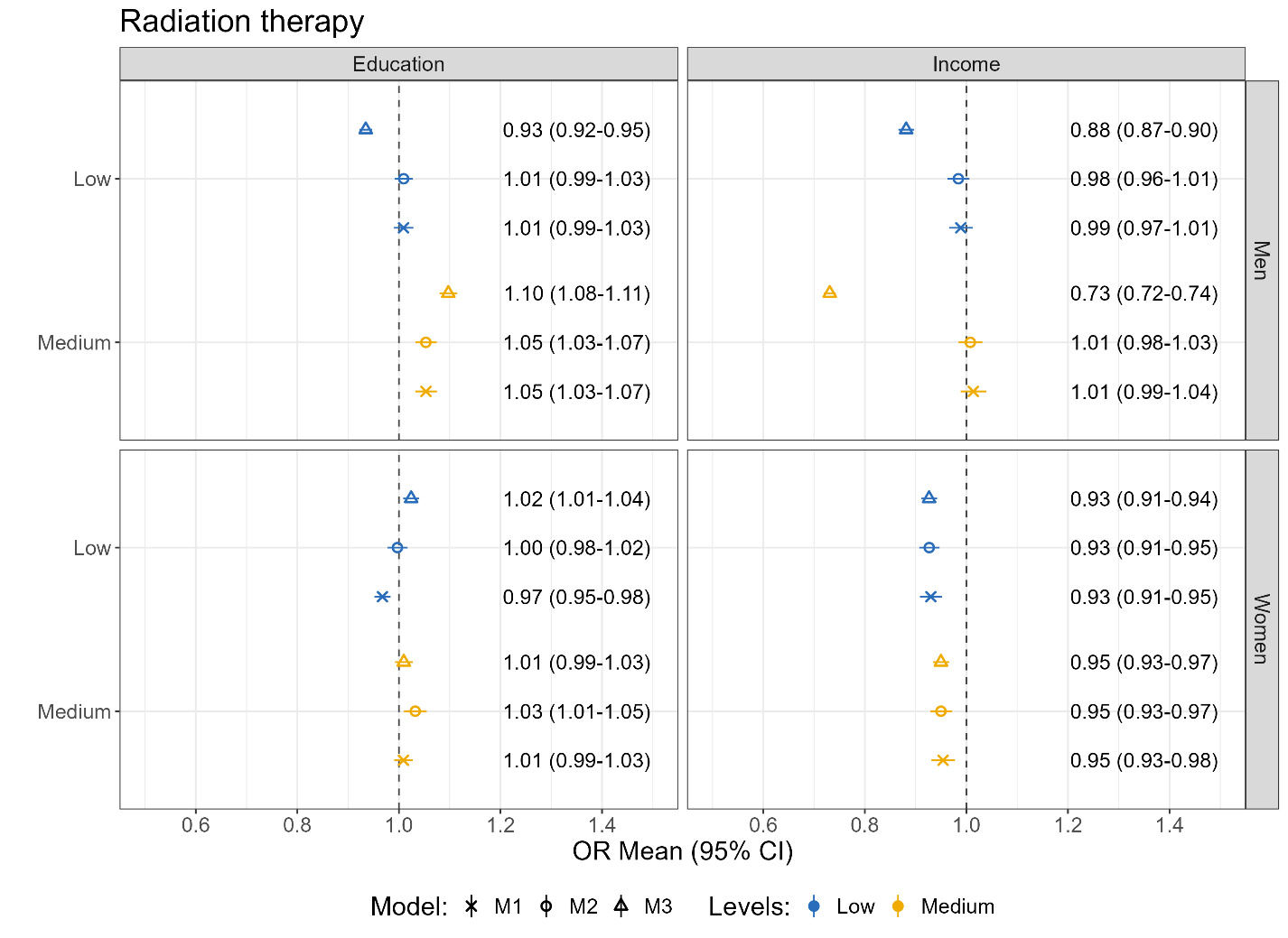
**

The outcome is taking radiation therapy or not. The figures show the mean OR and 95% CI of low or medium education (income) comparing with high education (income) estimated from logistic regression models. Covariates were adjusted in the models sequentially. The upper panels are for samples diagnosed with early stage cancers. The lower panels are for sample diagnosed with later stage cancers.

M1: logistic regression with education (or income), cancer type, stage at diagnosis, age at cancer diagnosis, year at diagnosis, year of birth, marital status, origin, region of residence and urbanization of the region.

M2: M1 + income (or education)

M3: M2 + education * income

### Supplementary Figure 14: Sensitivity analyses of SES factors on cancer-specific mortality for all cancers, stratified by sex

**
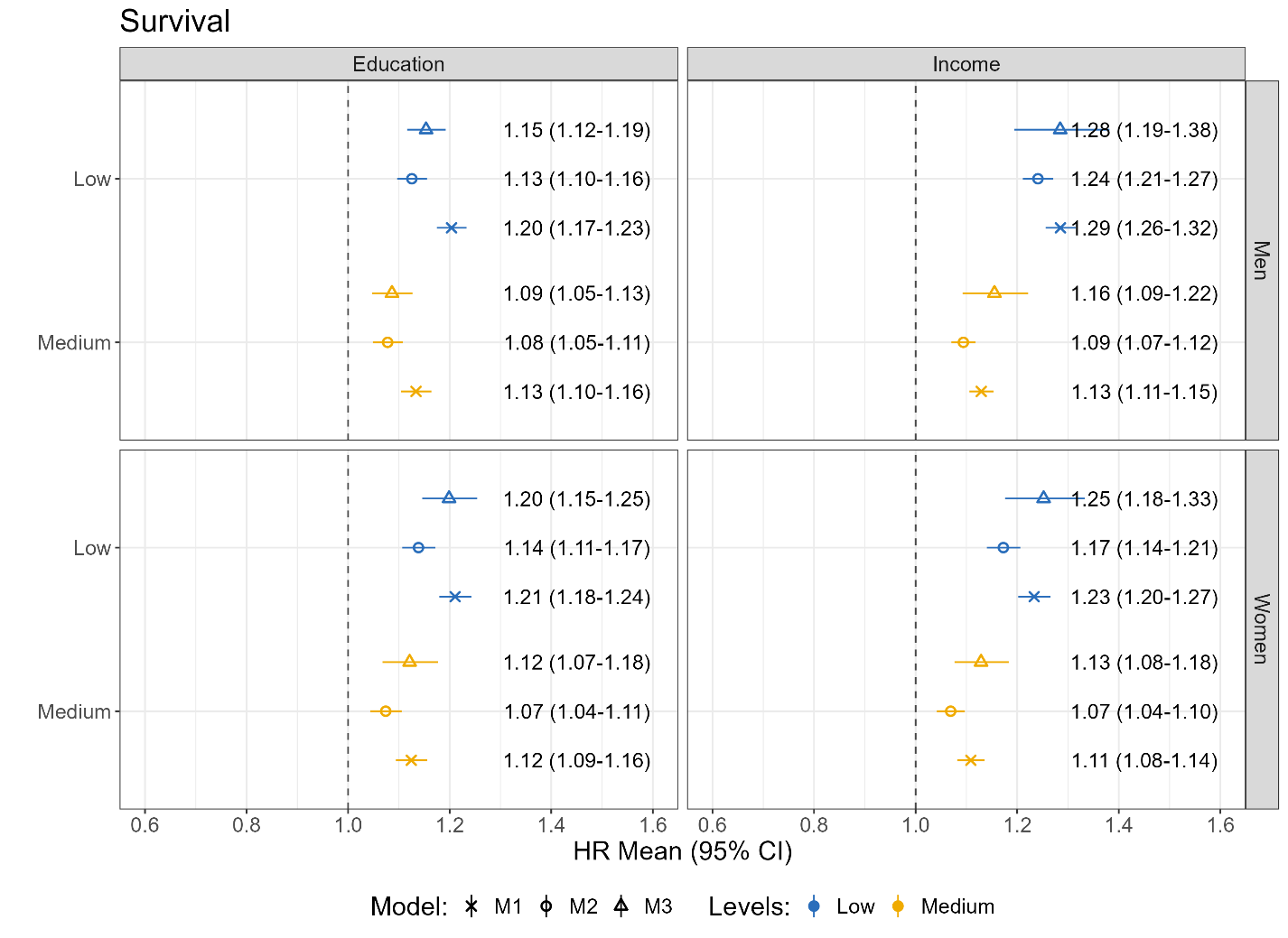
**

The outcome is the mortality of cancer patients. The figures show the mean HR and 95% CI of low or medium education (income) comparing with high education (income) estimated from Cox models. Covariates were adjusted in the models sequentially.

M1: Cox regression with education (or income), cancer type, stage at diagnosis, treatments, age at cancer diagnosis, year at diagnosis, year of birth, marital status, origin, region of residence and urbanization of the region.

M2: M1 + income (or education)

M3: M2 + education * income
